# Androgen-induced exosomal miR-379-5p release determines granulosa cell fate: cellular mechanism involved in polycystic ovaries

**DOI:** 10.1186/s13048-023-01141-1

**Published:** 2023-04-12

**Authors:** Reza Salehi, Brandon A. Wyse, Meshach Asare-Werehene, Fereshteh Esfandiarinezhad, Atefeh Abedini, Bo Pan, Yoko Urata, Alex Gutsol, Jose L. Vinas, Sahar Jahangiri, Kai Xue, Yunping Xue, Kevin D. Burns, Barbara Vanderhyden, Julang Li, Yutaka Osuga, Dylan Burger, Seang-Lin Tan, Clifford L. Librach, Benjamin K. Tsang

**Affiliations:** 1 Chronic Disease Program, Ottawa Hospital Research Institute, Ottawa, ON Canada; 2 Departments of Obstetrics and Gynecology, and Cellular and Molecular Medicine, University of Ottawa, Ottawa, ON Canada; 3 CReATe Fertility Centre, Toronto, ON Canada; 4 Cancer Therapeutics Program, Ottawa Hospital Research Institute, Ottawa, ON Canada; 5 Department of Animal BioScience, University of Guelph, Guelph, ON Canada; 6 Department of Obstetrics and Gynecology, University of Tokyo, Tokyo, Japan; 7 Division of Nephrology, Department of Medicine, Kidney Research Centre, University of Ottawa, Ottawa, ON Canada; 8 Department of Gynecology, The Affiliated Obstetrics and Gynecology Hospital, Nanjing Medical University, Nanjing Maternity and Child Health Care Hospital, Nanjing, Jiangsu Province, China; 9 Department of Obstetrics and Gynecology, McGill University, Montreal, QC Canada; 10 Originelle Fertility Clinic and Women’s Health Centre, Ottawa, ON Canada; 11 Departments of Obstetrics and Gynaecology, Physiology, Institute of Medical Sciences, University of Toronto, Toronto, ON Canada

## Abstract

Polycystic ovarian syndrome (PCOS) is a complex multi-factorial syndrome associated with androgen excess and anovulatory infertility. In the current study, we investigated the role of dihydrotestosterone-induced exosomal miR-379-5p release in determining the destiny of the developing follicles. Our hypothesis was that androgen regulates granulosa cell miR-379-5p content by facilitating its exosomal release in a follicular-stage dependent manner, a process which determines granulosa cell fate. Compared to human non-PCOS subjects, individuals with PCOS exhibit higher follicular fluid free testosterone levels, lower exosomal miR-379-5p content and granulosa cell proliferation. Androgenized rats exhibited lower granulosa cell miR-379-5p but higher phosphoinositide-dependent kinase-1 (PDK1; a miR-379-5p target) content and proliferation. Androgen reduced granulosa cell miR-379-5p content by increasing its exosomal release in preantral follicles, but not in antral follicles in vitro. Studies with an exosomal release inhibitor confirmed that androgen-induced exosomal miR-379-5p release decreased granulosa cell miR-379-5p content and proliferation. Ovarian overexpression of miR-379-5p suppressed granulosa cell proliferation, and basal and androgen-induced preantral follicle growth in vivo*.* These findings suggest that increased exosomal miR-379-5p release in granulosa cells is a proliferative response to androgenic stimulation specific for the preantral stage of follicle development and that dysregulation of this response at the antral stage is associated with follicular growth arrest, as observed in human PCOS.

**Supplementary Information:**

The online version contains supplementary material available at 10.1186/s13048-023-01141-1.

## Significance

Using human derived samples and rat PCOS models, as well as granulosa cell and follicle cultures, we tested the hypothesis that androgen regulates granulosa cell miR-379-5p content by facilitating its exosomal release in a follicular-stage dependent manner, a process which determines granulosa cell fate and follicle destiny. Our studies demonstrate for the first time that granulosa cell exosomal miR-379-5p release in response to androgenic stimulation is specific to the preantral stage of follicle development but absent during the antral follicle growth. Our studies shed light on the cellular basis for the androgen-induced regulation of ovarian follicular growth in follicular stage-dependent manner and how its dysregulation could result in antral follicular growth arrest in PCOS.

## Introduction

Polycystic ovarian syndrome (PCOS) is a multi-factorial syndrome with complex pathology. PCOS is associated with androgen excess, increased ovarian preantral follicular growth, antral follicle growth arrest and chronic anovulation [1]. Whereas a balanced level of androgen is important in normal ovarian follicular development [2–4], PCOS subjects exhibit androgen excess and suffer from anovulatory infertility [1]. Studies with cell-specific androgen receptor knockout mice, indicate that granulosa cells are the main target cells for androgen. Reduced preantral follicle development in these mice demonstrates that androgen signalling is mainly involved in preantral follicle development [5, 6], potentially through proteasomal degradation and reduced androgen receptors in antral follicle granulosa cells [7–9]. To study the pathological basis of PCOS, we and others have established a chronically androgenized rodent model with a relatively similar reproductive and metabolic phenotypes of human PCOS [9–17].

MicroRNAs (miRNAs) are small non-coding RNAs which play important roles in the regulation of various physiological and pathological processes by post-transcriptionally down-regulating target gene expression [18]. Abnormal miRNA expression has been linked to diseases, including PCOS [18–26]. We and others have demonstrated that androgen regulates cell fate (proliferation versus apoptosis) via up- or down-regulation of miRNAs [18, 26–30]. However, most miRNA functional studies have been conducted in cancer cells [31–34] and less is known about the roles of androgen-regulated miRNAs (e.g. miR-379-5p [18]) in reproductive endocrinopathies.

We have previously assessed the changes in the ovarian miRNA profile associated with PCOS in a androgenized rat PCOS model [18]. Our results indicate that 89 miRNAs, including miR-379-5p, are differentially expressed between dihydrotestosterone (DHT)-treated and control rats. MiR-379-5p is a member of the miRNA cluster DLK1-DIO3 [35]. Up-regulation of miR-379-5p in granulosa cells from subjects with premature ovarian insufficiency is associated with suppressed cell proliferation [36], suggesting that miR-379-5p is involved in ovarian follicular development. However, further studies are needed to determine its function and regulation during ovarian follicular growth and development.

Extracellular vesicles, including small (exosomes) and large (microvesicles) vesicles, are involved in cell–cell communication [37–39] by selectively packaging and transferring bioactive materials (e.g., miRNAs [40, 41]). Exosomes are vesicles of approximately 30–150 nm in size. These vesicles are formed within endosomes by membrane invagination. Microvesicles (size; 0.1 to 1.0 μm) are produced via membrane blebbing in response to cell stress [42]. It has been demonstrated that androgen attenuates follicular atresia through miR-125b-mediated suppression of granulosa cell pro-apoptotic protein expression [30], suggesting that granulosa cell miRNAs may have a direct regulatory role in ovarian follicular growth [43]. MiRNA profiling studies have identified several differentially expressed miRNAs in follicular fluid from PCOS women [25, 44, 45]. MiRNAs are present in both extracellular vesicles and extracellular vesicle-free media [44] and these miRNAs are regulators of reproductive, endocrine, and metabolic processes [25, 44]. Earlier hypotheses suggested that exosomes may function by expelling unusable cellular constituents from cells. However, the role of exosome secretion in regulating the cellular content of miRNAs and the function of exosome-secreting cells is largely unexplored.

In the current study, our specific objectives were to examine (1) the regulation of granulosa cell-extracellular vesicle release in response to androgen exposure; (2) if and how androgen regulates the cellular and extracellular content of miR-379-5p in granulosa cells; and (3) whether regulation of these processes is follicular stage-specific. Our overall hypothesis was that androgenic regulation of ovarian follicular development is follicular stage-specific, a phenomenon which is dependent on exosomal miR-379-5p release. In this study, we have demonstrated, for the first time, that androgen regulates ovarian exosome dynamics and their miRNA content in the control of follicle growth, and how this process may be compromised in PCOS. Our findings indicate that androgen regulates follicle growth in a stage-dependent manner via changes in the cellular and extracellular content of miR-379-5p, with phosphoinositide-dependent kinase-1 (PDK1) being its molecular target and responsible for the activation of the AKT pathway. Results from our animal studies were supported in part by our clinical observations that PCOS subjects at the preovulatory stage had lower exosomal miR-379-5p content and attenuated granulosa cell proliferation. These findings suggest that increased exosomal miR-379-5p release is a proliferation stimulus that is specific for preantral follicle granulosa cells, and this is subject to androgenic regulation.

## Results

### Human studies

#### Human PCOS subjects had a lower exosomal miR-379-5p content in follicular fluids and granulosa cell proliferation from large dominant follicles

To investigate the possible role of mir-379-5p in PCOS pathogenesis, its exosomal and extracellular vesicle-depleted follicular fluid content as well as granulosa cell proliferation in PCOS and non-PCOS subjects were compared. Dominant follicles (≥ 20 mm) of PCOS subjects had significantly higher ovarian follicular fluid free testosterone levels compared with that of non-PCOS. PCOS subjects also exhibited a lower exosomal miR-379-5p content (relative to miR-92a-3p, the content of which remained constant through the current study; Supplementary Fig. 1A) and granulosa cell proliferation (Supplementary Fig. 1B,C). MiR-379-5p was detected in follicular fluid depleted of extracellular vesicle, but its levels were not different from those from non-PCOS subjects (Supplementary Fig. 1A), suggesting that androgen excess in PCOS subjects dysregulates the association between cellular and exosomal content of miR-379-5p and subsequently cell proliferation in large dominant follicles.

### Rat studies

#### DHT reduced miR-379-5p and induced AKT pathway in rat granulosa cells

**Fig. 1:**
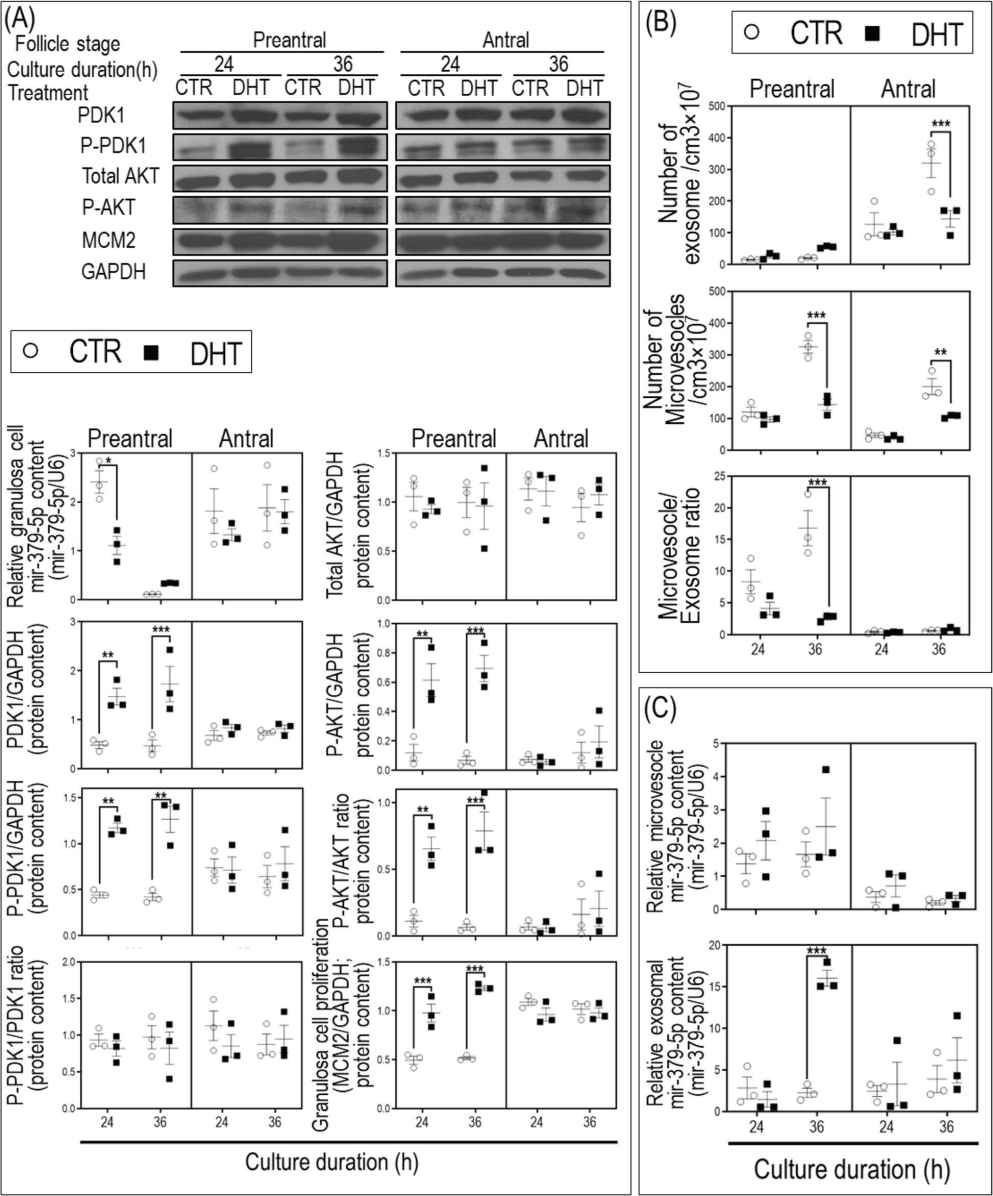
DHT reduces mir-379-5p content, increases its exosomal release and PDK1 content in granulosa cells from rat preantral but not antral follicle in vitro. **A** Reduced granulosa cell mir-379-5p content was associated with increased PDK1 and P-PDK1 protein levels and activation of Akt-mediated cell proliferation in preantral (increased P-AKT/GAPDH, P-AKT/AKT ratio and MCM2/GAPDH protein levels) but not antral follicle stage in vitro. **B** DHT treatment reduced microvesicles concentration at 36 h in both pre- and antral follicular stages. Exosomes concentration was reduced at 36 h post-DHT treatment in granulosa cells from antral follicles, but not in that of preantral follicle. Microvesicles/exosomes ratio was reduced at 36 h post-DHT treatment in granulosa cells from preantral follicles, but not in that of antral follicles. **C** DHT increased exosomal mir-379-5p level (normalized to U6), but not that of microvesicles in the conditioned medium in vitro. Granulosa cells were isolated from preantral follicles (Diethylstilbestrol-primed 21 day old rats; 1 mg/d, subcutaneous injection for 3 consecutive days) and antral follicles (Equine chorionic gonadotropin–injected 22-day old rats; 10 IU intraperitoneal injection, animals sacrificed 48 h post-injection) and cultured without or with ± DHT (1 µM, 24 h and 36 h). Exosomes and microvesicles were isolated from granulosa cell-conditioned medium by differential centrifugations, and sizing and concentration of extracellular vesicles were determined by nanoparticle tracking analysis. Protein extraction and Western blotting were performed as described previously [15]. Signal intensities generated on the film were measured densitometrically using Image J and normalized over that of GAPDH. U6 RNA was used to normalize miR-379-5p content. Results are expressed as means ± SEM (*n* = 3 replicates, each from 2 rats). Data were analyzed by three-way ANOVA and tukey post hoc. *P < 0.05; **P < 0.01, ***P < 0.001

To investigate if and how androgen excess regulates miRNA expression in granulosa cells, several candidate miRNAs (miR-24, miR-9, miR-379-5p, and let-7d) were selected based on our previous miRNA profile analysis of the whole ovary of androgenized rat PCOS model [18]. Expression of candidate miRNAs in granulosa cells isolated from immature rats and those treated with DHT for 24 h and 36 h in vitro were compared with controls. DHT significantly reduced the cellular content of miR-379-5p (Fig. 1A) but not that of miR-24, miR-9, let-7d (Supplementary Fig. 2). Using the bioinformatics website (TargetScan and RNA22 v2 microRNA target detection) and based on published findings [33], transforming growth factor beta receptor I (TGFBR1) and phosphoinositide-dependent kinase-1 (PDK1, responsible to activate AKT by phosphorylation of Thr 308) were identified as potential downstream targets of miR-379-5p. PDK1 protein content was significantly increased 24 h and 36 h post-DHT treatment and was associated with higher granulosa cell proliferation, as evidenced by increased MCM2 (Minichromosome Maintenance Complex Component 2) content (Fig. 1A). However, TGFBR1 protein content was not affected by DHT treatment (Supplementary Fig. 3). Therefore, we focused on PDK1 as a potential target gene regulated by miR-379-5p in subsequent studies.

#### Androgen-induced exosomal miR-379-5p release reduces its granulosa cell content, resulting in higher PDK1-mediated cell proliferation in rat preantral but not in antral follicles

DHT significantly reduced granulosa cell miR-379-5p content 24 h post-treatment at the preantral but not in at the antral follicle stage (Fig. 1A). The reduction of granulosa cell miR-379-5p content was accompanied by increases in PDK1 and P-PDK1 content at 24 h and 36 h in preantral follicles, but not in antral follicle in vitro (Fig. 1A). Moreover, DHT treatment significantly increased P-AKT, P-AKT/AKT ratio and proliferation (MCM2, Fig. 1A) in preantral but not antral follicle granulosa cells.

To investigate if changes in granulosa cell miR-379-5p content is regulated by changing its cellular synthesis or through its packaging and release from cell, and whether these responses are follicular stage-specific, we assessed cellular content of pri-miR-379 (precursor of miR-379-5p), the number of microvesicles, exosomes and their miR-379-5p content in the conditioned medium from preantral and antral follicle granulosa cells at 24 h and 36 h post-DHT treatment in vitro. The baseline levels of pri-miR-379-5p were higher in the antral follicle granulosa cells compared with that of the preantral regardless of the time of culture (Supplementary Fig. 4). DHT increased granulosa cell pri-miR-379 content (Supplementary Fig. 4) in both preantral and antral granulosa cells, indicating that miR-379-5p is an androgen-responsive miRNA. Antral follicle granulosa cells also had higher cellular pri-miR-379 content post-DHT treatment than that of preantral follicle stage. However, there were no significant interactions between follicle stage and DHT treatment (stage x DHT) nor between follicle stage, DHT treatment and culture duration (stage x DHT treatment x culture duration). DHT treatment had no influence on the size of granulosa cell-derived microvesicles and exosomes irrespective of follicle stage or culture duration (Supplementary Fig. 5). DHT treatment for 36 h reduced microvesicle release from both preantral and antral follicular granulosa cells (Fig. 1B), and reduced exosome release in antral but not preantral follicle granulosa cells (Fig. 1B). The ratio of microvesicle to exosome released was reduced 36 h post-DHT treatment in preantral follicle granulosa cells (Fig. 1B). miR-379-5p content in exosomes but not in microvesicles was increased 36 h following exposure of preantral follicle granulosa cells with androgen in vitro (Fig. 1C). DHT had no significant influence on the microvesicle and exosomal content of miR-379-5p from antral follicle granulosa cells (Fig. 1C). miR-379-5p was not detectable in extracellular vesicle-depleted conditioned media of preantral and antral follicular granulosa cells (data not presented). These results suggest that preantral follicle granulosa cells selectively package and release miR-379-5p through exosomes in response to androgen, rather than increasing the number of exosome release.

To determine if androgen regulates exosomal miRNA release and if this response is miR-379-5p-specific, exosomal miR-24, miR-9, and let-7d contents were also assessed in preantral follicle granulosa cell cultures for 24 h and 36 h in the absence or presence of DHT. Our results indicate that DHT treatment did not affect their exosomal contents, suggesting that the androgenic regulation of exosomal miRNA contents in preantral granulosa cells is miR-379-5p-specific (Supplementary Fig. 2).

#### Androgenized rats had lower granulosa cell miR-379-5p content and higher cellular PDK1 content and proliferation

**Fig. 2:**
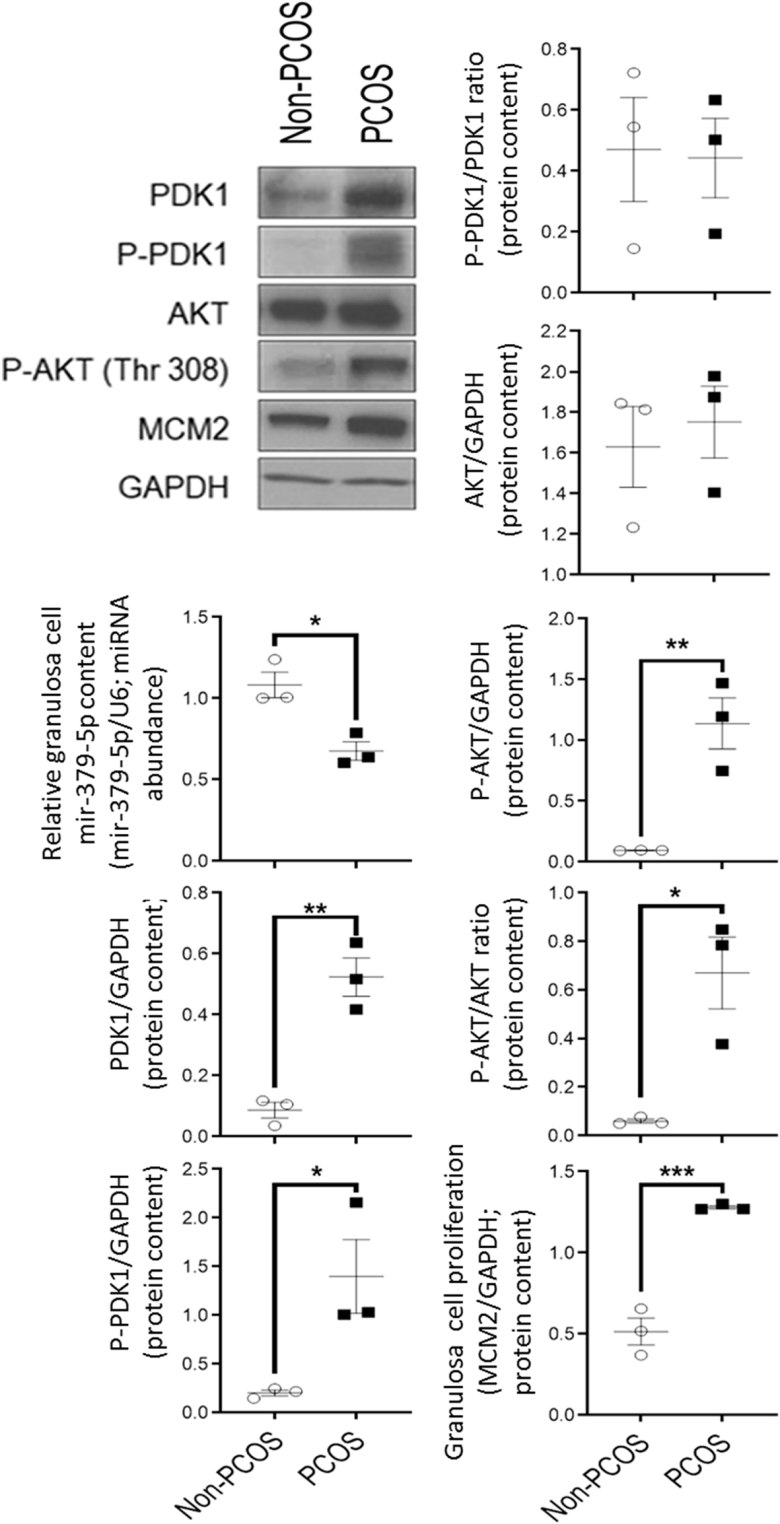
Reduced granulosa cell mir-379-5p content is associated with increased PDK1-mediated cell proliferation in an androgenized PCOS rat model. Lower cellular mir-379-5p content in dihydrotestosterone (DHT)-induced PCOS rat model was associated with increased granulosa cell protein content of PDK1 and P-PDK1, and P-PDK1/PDK1 ratio. Increased PDK1 protein content was accompanied by activation of Akt pathway (increased P-Akt/Akt ratio) and increased granulosa cell proliferation (higher MCM2 protein content). Granulosa cells were isolated from 21 day old rats randomly divided into two experimental groups (Non-PCOS and PCOS), and subcutaneously implanted for 28 days with a capsule without and with DHT respectively, as per Materials and Methods. Protein extraction and Western blotting were performed as described previously [15]. Signal intensities generated on the film were measured densitometrically using Image J and normalized over that of GAPDH. U6 RNA was used to normalize miR-379-5p content in granulosa cells. Results are expressed as means ± SEM (*n* = 3 replicates, each from 1 rat). Data were analyzed by Student “t” test. *P < 0.05, **P < 0.01

To investigate if androgen excess is associated with down-regulation of granulosa cell miR-379-5p content in vivo, its expression was evaluated in granulosa cells isolated from preantral follicles of androgenized PCOS rats. One month of DHT treatment in vivo significantly reduced granulosa cell miR-379-5p content (*P* < 0.05; Fig. 2). This response was associated with increased PDK1 and P-PDK1 contents (Fig. 2). Although total AKT content was not altered by androgen treatment in vivo, DHT-treated rats exhibited higher P-AKT (Thr308) levels and a higher P-AKT/AKT ratio (Fig. 2). Furthermore, activation of the AKT pathway was accompanied by increased granulosa cell proliferation, as indicated by an increased cellular MCM2 content in granulosa cells from DHT-treated rats (Fig. 2).

#### miR-379-5p regulates rat granulosa cell proliferation through PDK1 suppression

**Fig. 3:**
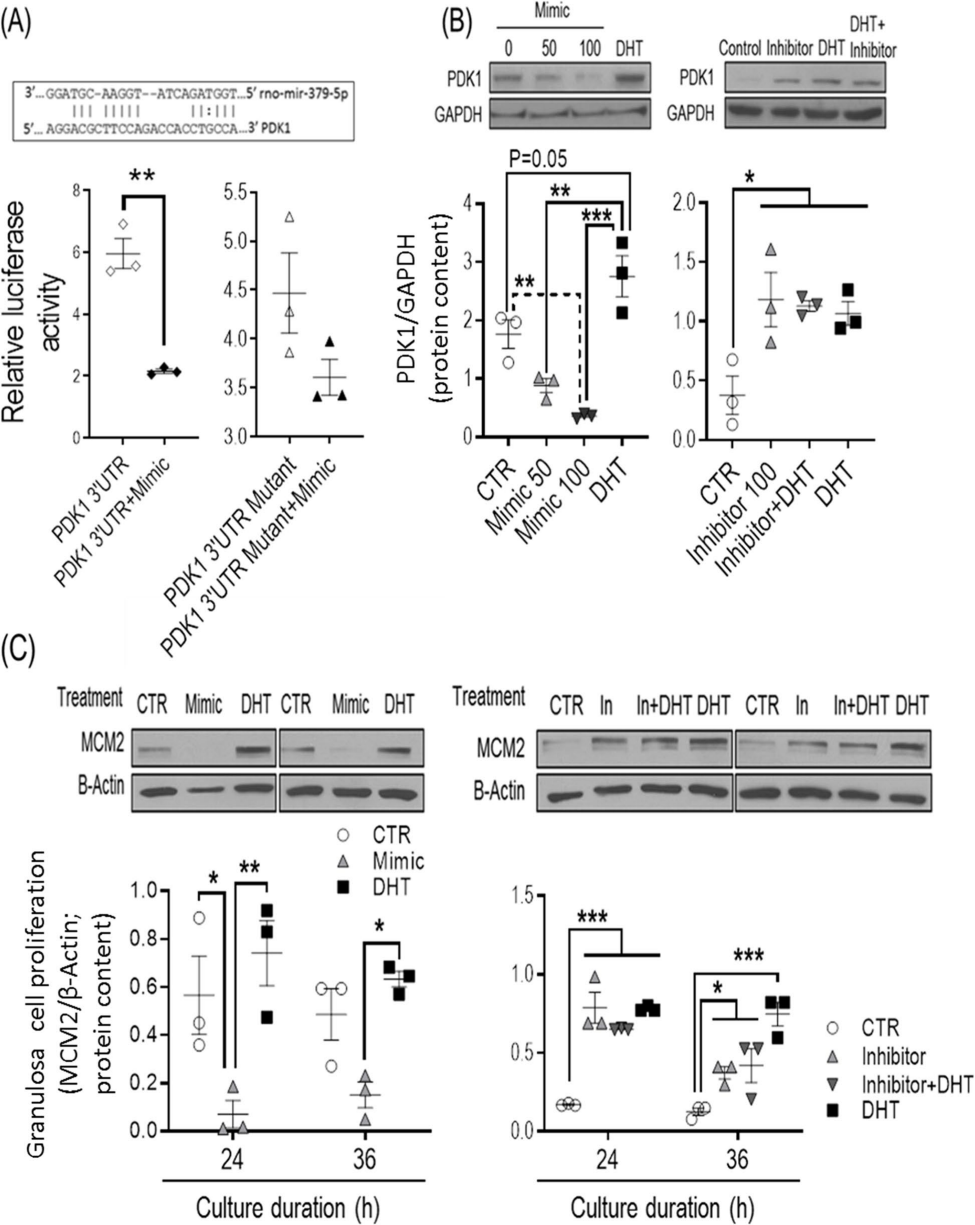
mir-379-5p regulates rat granulosa cell proliferation through PDK1 suppression in vitro. **A** Binding of mir-379-5p mimic to PDK1 3’UTR mRNA, but not to its mutant (binding site deleted) significantly reduced luciferase activity; **B** & **C** mir-379-5p mimic reduced PDK1 protein content and cell proliferation (MCM2 protein content) but, miR-379-5p inhibitor, like DHT, increased these responses in vitro. Granulosa cells were isolated from preantral follicles (Diethylstilbestrol-primed 21-day old rats; subcutaneous injection for 3 consecutive days) and either transfected with (**A**) the reporter vector containing a complementary mir-379-5p sequence in the 3ʹ-UTR of the PDK1 (or its mutant as control) or (**B** & **C**) with mir-379-5p mimic (mirVana Mimics, MC10316, Thermo-Fisher) and inhibitor (mirVana inhibitor, MH10316) for 24 h before culture without or with DHT (1 µM; 24 h and 36 h). Protein extraction and Western blotting were performed as described previously [15]. Signal intensities generated on the film were measured densitometrically using Image J and normalized over that of GAPDH or β-Actin. Results are expressed as means ± SEM (*n* = 3 replicates, each from 2 rats). Data were analyzed by t-test: **P < 0.001 (**A**); one-way ANOVA & tukey post hoc (**B**); three-way ANOVA and tukey post hoc (**C**). *P < 0.05; **P < 0.01, ***P < 0.001

To determine whether miR-379-5p directly regulates PDK1 expression, we first examined its binding to the 3ʹ UTR section of PDK1 mRNA, using a dual-reporter luciferase assay with a reporter vector containing a complementary miR-379-5p sequence in its 3ʹ-UTR (Fig. 3A). The binding of miR-379-5p mimic to the PDK1 3′-UTR, but not to its mutant (binding site deleted; Fig. 3A), significantly reduced luciferase activity in preantral follicle granulosa cells transfected with the reporter vector, confirming binding of the miRNA to the PDK1 mRNA (Fig. 3A). Further studies with miR-379-5p mimic and inhibitor demonstrate that miR-379-5p mimic significantly suppressed granulosa cell PDK1 protein content and proliferation (Fig. 3B,C), two down-stream responses readily attenuated by its inhibitor (Fig. 3B,C), suggesting that miR-379-5p reduces granulosa cell proliferation through PDK1 suppression.

#### miR-379-5p exosomal release is a cellular mechanism through which androgen up-regulates granulosa cell PDK1 content and proliferation in rat preantral follicles

**Fig. 4:**
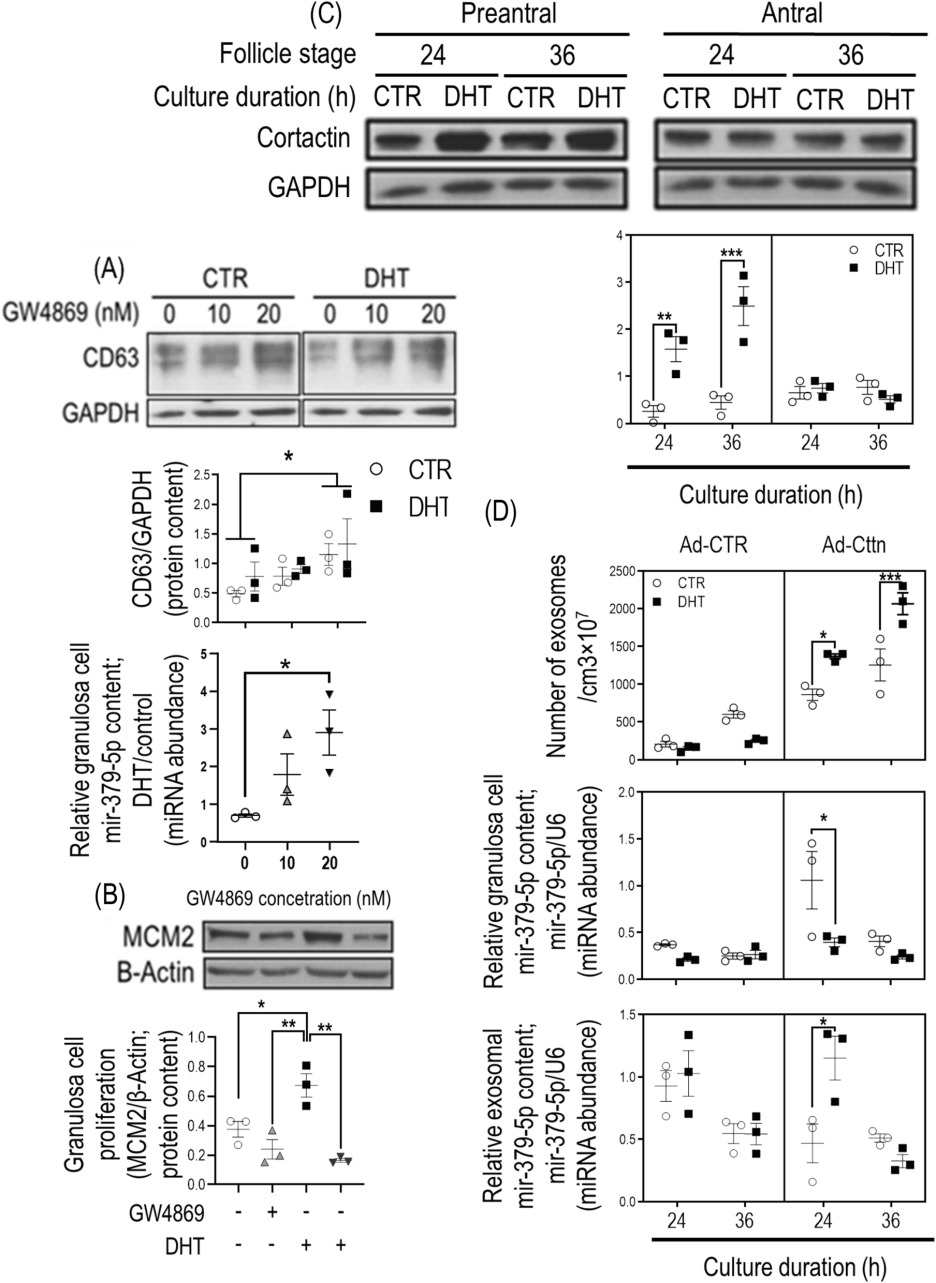
Androgen-induced exosomal mir-379-5p release as a determinant of rat granulosa cell mir-379-5p content and proliferation. **A** & **B** Irrespective of DHT treatment, GW4869 at 20 nM effectively inhibited exosome release, as evident by increased granulosa cellular content and reduced conditioned medium level of CD63 protein (exosome marker; Supplementary Fig. 3). The inhibition of exosome release increased granulosa cell mir-379-5p content (normalized to U6; DHT/control ratio) and down-regulate cell proliferation (MCM2) induced by DHT; **C** DHT increased granulosa cell cortactin protein content in granulosa cells from preantral, but not antral follicle; **D** cortactin overexpression in combination with DHT significantly increased exosome release in antral follicle granulosa cells and reduced granulosa cell mir-379-5p content (normalized to U6). Granulosa cells were isolated from preantral follicles (Diethylstilbestrol-primed 21-day old rats;1 mg/d, subcutaneous injection, for 3 consecutive days) or antral follicles (Equine chorionic gonadotropin–injected 21-day old rats; 10 IU, intraperitoneal injection, animals sacrificed 48 h post-injection). Exosome release was inhibited with GW4869 pre-treatment (0, 10 and 20 mM, 24 h) before DHT (0 to 1 µM, 24 h and 36 h). Exosomes were isolated from granulosa cell conditioned medium by differential centrifugations. To induce exosome release in antral follicle stage, granulosa cells were infected with adenoviral sense full-length cortactin cDNA or adenoviral control vector at 50 MOI (24 h), and then cultured with DHT (0 to 1 µM, 24 h and 36 h). Protein extraction and Western blotting were performed as described previously [15]. Signal intensities generated on the film were measured densitometrically using Image J and normalized over that of GAPDH or β-Actin. U6 RNA was used to normalize miR-379-5p content. Results are expressed as means ± SEM (*n* = 3 replicates, each from 2 rats). Data were analyzed by (**A** & **B**) two-way ANOVA and tukey post hoc; (**C** & **D**) three-way ANOVA and tukey post hoc. *P < 0.05; **P < 0.01, ***P < 0.001

The role of exosomal miR-379-5p release on its cellular content and those of PDK1 was studied in vitro*,* using the exosome release inhibitor GW4869 (chemical inhibitors of N-sphingomyelinase, nSMase) [46, 47]. To determine the optimal concentration of GW4869 for inhibition of exosome release, preantral follicle granulosa cells were pre-treated with different concentration of GW4869 (0, 10 and 20 nM) for 24 h and then cultured in the absence and presence of DHT (1 µM; 36 h). Concentration–response studies indicate GW4869 treatment at 20 nM significantly suppressed basal and DHT (1 μM; 36 h)-induced exosome release from preantral follicle granulosa cells, as evidenced by increased granulosa cell CD63 content (exosome marker; Fig. 4A), and its reduction in culture conditioned medium (Supplementary Fig. 6). Inhibition of exosome release increased granulosa cell miR-379-5p content in the presence of DHT (Fig. 4A). To further investigate if exosomal miR-379-5p release regulates granulosa cell proliferation, preantral follicle granulosa cells were pre-treated with GW4869 (20 nM; 24 h), and then treated with or without DHT (1 µM; 36 h). Inhibition of exosome release suppressed DHT-induced granulosa cell proliferation (Fig. 4B), supporting our hypothesis that exosomal miR-379-5p release is a regulatory mechanism by which androgen increases granulosa cell proliferation in preantral follicles.

#### The induction of exosome release reduces granulosa cell miR-379-5p content in the rat antral follicle stage

Cortactin is involved in exosome docking and release and its overexpression induces exosome release without affecting its content [48]. In contrast to the more responsive granulosa cells from preantral follicles, DHT failed to influence granulosa cell cortactin content (Fig. 4C) and proliferation in antral follicles in vitro, suggesting that the failure of androgen to induce granulosa cell proliferation in antral follicles could be due to dysregulated or non-responsive granulosa cell cortactin-mediated exosome release. To test this possibility, cortactin was overexpressed in granulosa cells from antral follicles to determine if cortactin-induced exosome release increases exosomal miR-379-5p release and reduces its cellular content. The overexpression of cortactin increased exosome release by antral follicular granulosa cell 24 h and 36 h post-DHT treatment in vitro (Cttn + DHT; Fig. 4D). Exosome release induction in Cttn + DHT increased exosomal miR-379-5p release and reduced its cellular content at 24 h (Fig. 4D), suggesting that the failure of androgen to induce granulosa cell proliferation in antral follicles could be due to lack of cortactin activation and miR-379-5p exosomal release.

#### Inhibition of exosome release suppresses DHT-induced rat preantral follicular growth

**Fig. 5:**
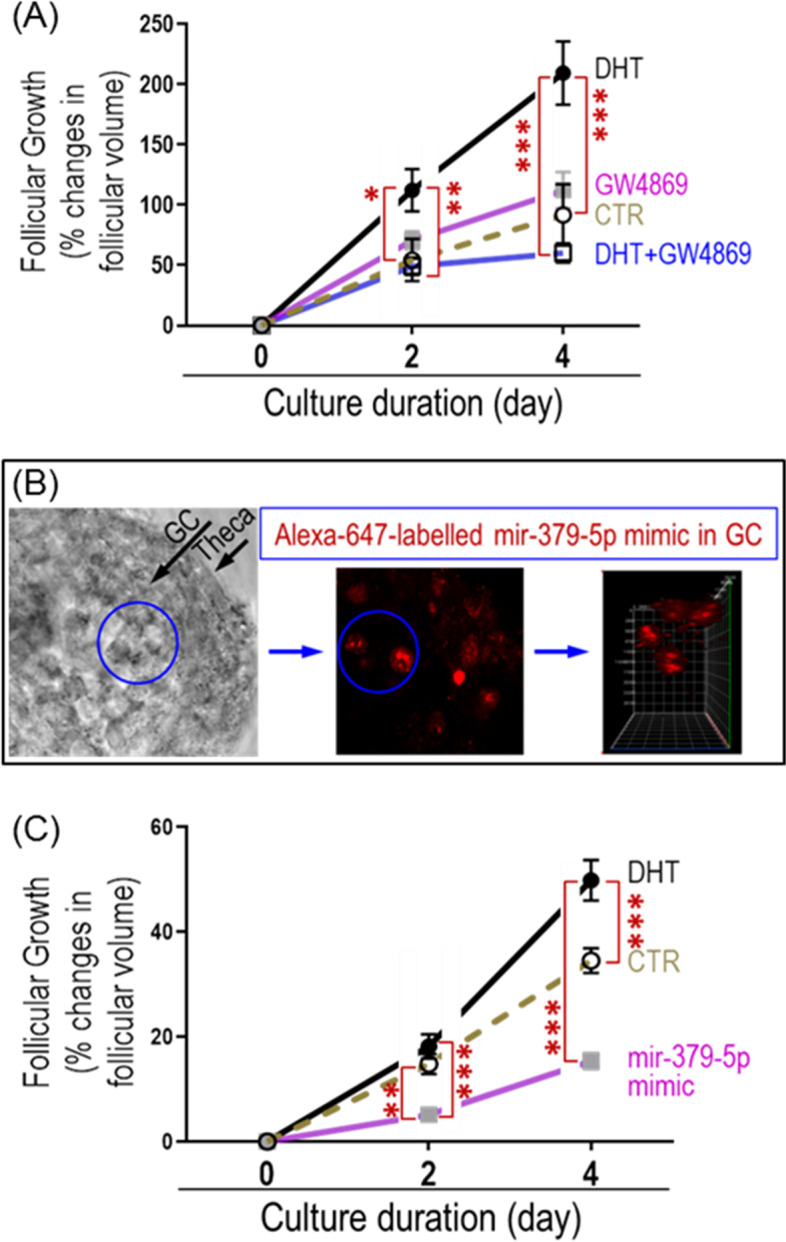
mir-379-5p suppresses rat preantral follicular growth. **A** Inhibition of exosomal release suppressed DHT-induced follicular growth compared to DHT and control on day 2 and 4 of culture. There were no difference in follicular volume between control and GW4869. **B**, **C** Transfection of preantral follicle with mir-379-5p mimic significantly suppressed the follicle growth compared to control and DHT. Alexa-647-labelled mir-379-5p mimic was used to confirm successful transfection of granulosa cells in preantral follicle. Preantral follicles (110 to 150 mm diameter) were mechanically isolated from 13- to 14-day old rats and individually cultured in 96 well dish. Exosome release was inhibited with GW4869 treatment (20 mM) for 24 h before DHT treatment (0 to 1 µM, 4 days). Preantral follicles was transfected with mir-379-5p mimic and cultured without or with DHT (1 µM, 4 days), and follicular growth was assessed on day 2 and 4 post-treatment. Results are expressed as change in mean follicular volume ± SEM (*n* = 15 follicles each). Data were analyzed by two-way ANOVA and tukey post hoc. *P < 0.05; **P < 0.01, ***P < 0.001

To determine if androgen-induced preantral follicular growth is mediated through exosomal miR-379-5p release, preantral follicles pre-treated with and without GW4869 (20 nM; 24 h) were cultured in the absence and presence of DHT (1 µM). Follicular growth in the four experimental groups: control, GW4869, DHT and DHT + GW4869 was assessed on days 2 and 4 post-DHT treatment. Our results indicate that, although there was no difference in follicular volume between control and GW4869 on days 2 and 4, inhibition of exosomal release suppressed the DHT-induced follicular growth (DHT + GW4869) compared to control, GW4869 or DHT treatment, alone (Fig. 5A).

#### MiR-379-5p suppresses the follicular growth of rat preantral follicles

To determine the role of miR-379-5p in follicular growth, preantral follicles were transfected with miR-379-5p mimic and cultured with or without DHT (1 µM), and follicular growth in vitro was assessed on days 2 and 4 post-treatment. Alexa-647-labelled miR-379-5p mimic was used to confirm successful transfection of granulosa cells in preantral follicles. Image analysis of the transfected preantral follicles indicates that a subset of granulosa cells was successfully transfected with Alexa-647-labelled miR-379-5p mimic (Fig. 5B), which significantly suppressed the follicle growth compared to that of control and DHT groups (Fig. 5C).

#### Reconstitution of DHT-treated rat granulosa cells with miR-379-5p-enriched exosomes attenuates the DHT-induced proliferative response in vitro

As we showed earlier, DHT-induced exosomal miR-379-5p release reduces its granulosa cell content and increases PDK1 content and proliferation in rat preantral follicle granulosa cells in vitro. In addition to the increased exosomal miR-379-5p release in response to androgen, the decreased granulosa cell miR-379-5p content could be due to decreased exosome uptake. To address this possibility, we investigated whether DHT alters granulosa cell exosome uptake by assessing if reconstitution of DHT-treated granulosa cells (miR-379-5p down-regulated) with miR-379-5p-enriched exosomes (DHT-exo) could attenuate the DHT-induced proliferative response in vitro (Supplementary Fig. 7A). Proliferation assays indicate that androgen increased cell proliferation in granulosa cells treated with exogenous control exosomes (CTR-exo). However, this response was attenuated by reconstitution of miR-379-5p by utilizing exosome-enriched in miR-379-5p isolated from DHT-treated donor granulosa cells (DHT-exo; Supplementary Fig. 7B,C). Exosome uptake was assessed by flow cytometry. Our results indicate that, while exosomes were effectively taken up by recipient granulosa cells, this response was not affected by DHT treatment of either donor or recipient granulosa cells (Supplementary Fig. 7C). This suggests that, despite the stimulatory role of androgen on granulosa cell exosomal miR-379-5p secretion, it plays minimal or no role in the regulation of exosome uptake in recipient cells.

#### MiR-379-5p overexpression suppresses the basal and androgen-induced rat preantral follicle growth by reduced granulosa cell proliferation

**Fig. 6:**
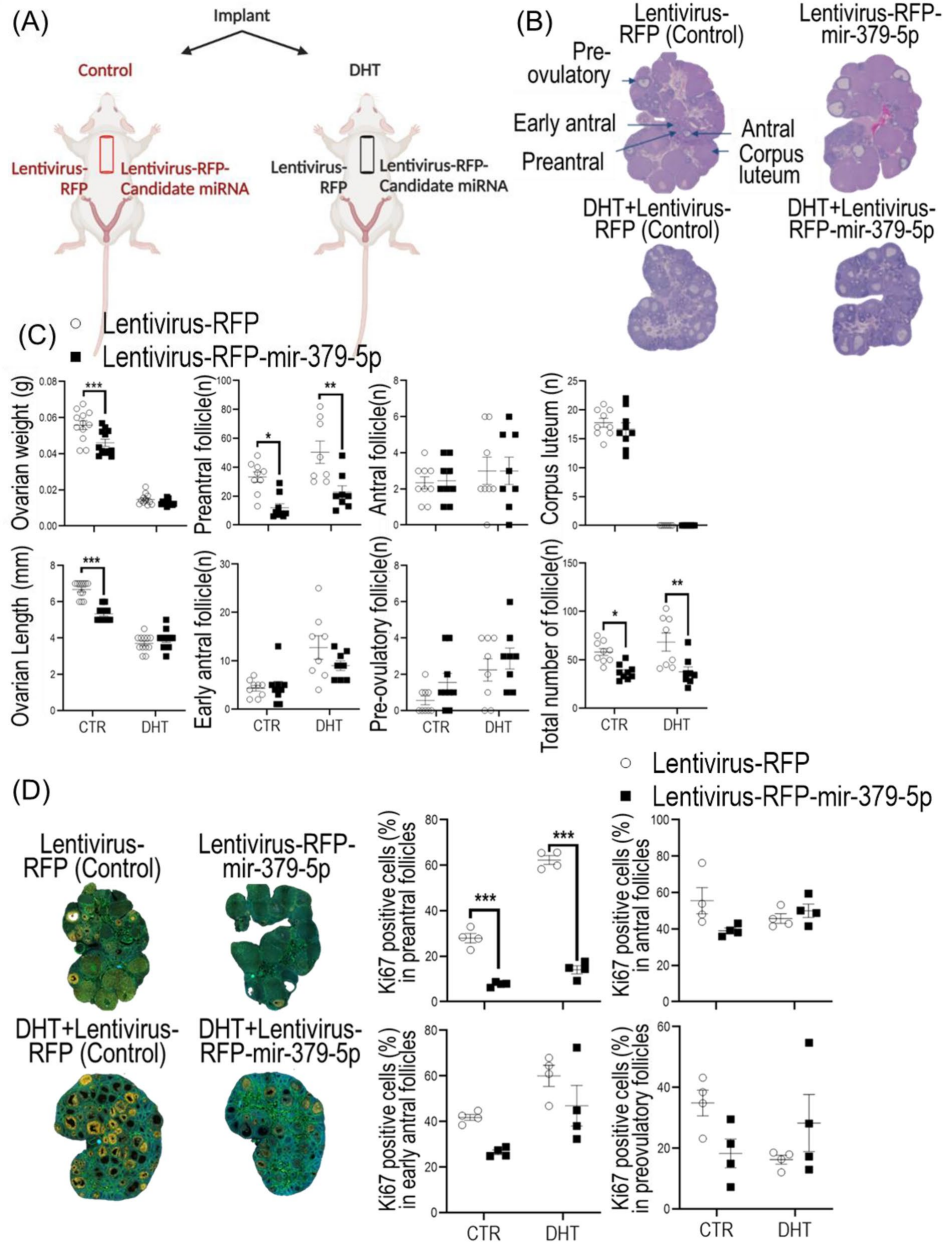
mir-379-5p suppresses the basal and androgen-induced rat preantral follicle growth through reduced granulosa cell proliferation in vivo. **A** A schematic image of experimental design and how rats received different treatments; **B** Histological images of ovaries which received the following treatments, lentivirus-RFP, lentivirus-RFP-mir-379-5p, DHT + lentivirus-RFP, DHT + lentivirus-RFP-mir-379-5p; **C**,** D **&** E** Ovarian mir-379-5p overexpression reduced ovarian growth (length and weight) in control group and suppressed granulosa cell proliferation and preantral follicle number in both control and androgenized rats. Lentivirus particles (2 μl) were injected under the ovarian bursa. The dosage and successful transduction was confirmed in Supplementary Fig. 10. *P < 0.05; **P < 0.01, ***P < 0.001

To further examine the role of miR-379-5p in follicular growth in vivo and whether miR-379-5p overexpression could suppress DHT-induced preantral follicle development, lentiviral miR-379-5p mimic was introduced under the ovarian bursa of control and androgenized rats (Fig. 6A). Ovarian growth (weight and length), follicular stage, number of follicles and their cell proliferation (Ki67) at each stage were compared between sham-control and DHT-treated rats. Our results indicate that ovarian miR-379-5p overexpression suppressed ovarian growth (length and weight; Fig. 6B,C) in control rats. In addition, ovarian miR-379-5p overexpression reduced the number of preantral follicle (Fig. 6B,C) and their granulosa cell proliferation (Fig. 6D,E) and total number of follicles (Fig. 6B,C) in both control and androgenized rats. These results provide further support that miR-379-5p is mainly involved in the growth and development of preantral follicles.

## Discussion

Androgen promotes both follicular growth and atresia in a follicular stage-dependent manner [6, 9, 11, 49] possibly through changes in the cellular content of miRNAs [18]. Emerging evidence suggests that exosomes contribute to many aspects of reproductive physiology and disorders through intercellular communication [42, 47]. However, the role of exosome secretion in determining miRNA content, and their down-stream consequences, in exosome-secreting cells remains relatively unexplored. In the current study, we have demonstrated if and how androgen-induced exosomal miR-379-5p release in granulosa cells determines the destiny of a growing follicle. Our results indicate that androgen regulates follicular growth in a stage-dependent manner, and this is via differential changes in cellular and extracellular content of miR-379-5p. Mechanistic studies indicate that PDK1, responsible for phosphorylation and activation of AKT, is a downstream target of miR-379-5p; and that androgenic stimulation of preantral follicles increases exosomal miR-379-5p release and reduces its granulosa cell content, resulting in enhanced PDK1-mediated granulosa cell proliferation. These responses appear to be specific to preantral follicles, since DHT failed to stimulate proliferation of granulosa cells from antral follicles, nor did it increase exosomal miR-379-5p release or decrease granulosa cell content. These findings may offer important mechanistic insight into developmental differences in the follicle growth dynamics associated with PCOS, a condition often associated with androgen excess.

Exosomes play important roles in intercellular communication by serving as vehicles for transferring various cellular constituents, such as proteins, lipids and nucleic acids, between cells. However, very little is known about the role of exosome release in determining the cellular miRNA content from exosome-releasing cells. It has been suggested that exosome release plays a crucial role in maintaining cellular homeostasis in exosome-releasing cells and inhibition of this process induces reactive oxygen species-dependent DNA damage and apoptosis in normal human cells [50]. In the present studies, we have demonstrated that androgen upregulates exosomal miR-379-5p release and proliferation of rat granulosa cells from preantral, but not antral follicles. The androgen-induced miR-379-5p release from preantral granulosa cells appears to be specific to miR-379-5p and is mediated by exosomes but not by microvesicles or simple diffusion (evident by its absence in extracellular vesicle-depleted conditioned medium). This may represent an androgen-induced proliferative mechanism to reduce its cellular content in preantral follicle granulosa cells. Our findings support the concept that miRNAs are selectively packaged into exosomes [51–53]. Based on an miRNA’s motif sequence, RNA-binding proteins can bind to the specific miRNA molecules, and assist the miRNA sorting process into exosomes. Heterogeneous nuclear ribonucleoprotein A2B1 (hnRNPA2B1) is one such RNA-binding protein capable of targeting specific miRNAs to control their sorting and loading into exosomes [54]. Recently, it has been shown that hnRNPA2B1 protein recognizes and specifically packages miRNA-17 and -93 into exosomes through binding into their AGG/UAG motifs [55, 56]. These binding motifs could provide a potential mechanism whereby hnRNPA2B1 exerts regulatory control over miRNA sorting. Interestingly, we also identified that miR-379-5p has a similar motif sequence and hence hnRNPA2B1 may be involved in selective packaging of miR-379-5p into exosomes in response to androgen. However, further investigations are required to confirm if this is indeed the case. Our current findings support the notion of exosomal packaging of miR-379-5p as a key regulator of granulosa cell proliferation. Specifically, inhibiting exosomal release with GW4869 during preantral follicular development resulted in the accumulation of intracellular miR-379-5p, leading to reduced granulosa cell proliferation.. Collectively, these results suggest that it is not the release of exosomes, but increased exosomal content of miR-379-5p released from granulosa cells in response to androgenic stimulation is a proliferative mechanism specific for the preantral stage of follicle development.

The reason (s) for the observed reduction in the granulosa cell content of mir-379-5p in the control after 36 h in the preantral stage is not known, but could be due to several factors including differential miRNA turnover in preantral and antral follicles and packaging of miR-379-5p into alternative extracellular vesicles not assessed in this study. miRNA turnover and degradation are a highly contested topic in the literature with studies reporting wide ranges of stability (28 h – 220 h) [57]. Furthermore, these dynamic changes are dependent on cell type, stress response, culture conditions, to name a few. In addition to altered stability, selective packaging of miR-379-5p into other extracellular vesicles not assessed in this study is possible, which is a limitation due to sample input constraints. Thus, the storage and packaging of cargoes in cells could be changed under certain conditions. It’s possible that the packaging of miRNA could be redirected from exosomes at 24 h to another vesicle type at 36 h. This, along with possible altered turnover dynamics, potentially explains why miR-379-5p content in the preantral stage granulosa cell decreased at 36 h with no increase in the exosomal miR-379-5p content. However, further studies are required to determine the potential pathway(s) involved in this mechanism. The androgen receptor (AR) plays important regulatory roles in ovarian follicular development and AR cellular content determines the capability of the target cell to respond to androgen stimulation. Ring finger protein 6 (RNF6), a member of E3 ligase family, induces AR ubiquitination [9]. In previous studies, we have observed that androgen induced ovarian follicular growth is dependent on RNF6-mediated, lysine site-specific, AR polyubiquitination, thus determining AR transcriptional activity, stability and abundance. Also, the androgenized rat PCOS model exhibits increased RNF6-mediated AR ubiquitination (K48), enhanced AR degradation, and decreased granulosa cell proliferation in the antral but not preantral follicle stage [9, 11]. In this study, and in contrast to the more responsive preantral follicle granulosa cells, granulosa cells in antral follicles failed to respond to DHT with increased cortactin expression and proliferation in vitro. On the other hand, cortactin overexpression in the antral follicle stage, increases its exosomal miR-379-5p release and reduces granulosa cell content in response to androgen, suggesting that the failure of androgen-induced granulosa cell proliferation in antral follicle could be due to AR degradation, lack of cortactin activation and miR-379-5p exosomal release. In preantral follicle stage, whereas DHT significantly increased cortactin protein content, it did not change that of exosome release. Therefore, exosome release may possibly be regulated differentially in preantral and antral follicle stages.

The content of cellular constituents, such as miRNAs and proteins, is determined by a balance between exosome release and uptake. Preferential uptake of extracellular vesicles derived from follicular fluid of small antral follicles, compared to those isolated from large follicles, stimulated granulosa cell proliferation through Src, PI3K/AKT, and MAPK signaling pathways [58]. The proliferative response could be explained by both the differential cellular contents and by the preferential uptake of extracellular vesicles [58]. In the current study, androgen treatment of donor and recipient cells did not alter exosome uptake. However, exosomal uptake was not assessed between different follicle stages, an aspect being considered for future studies. Although exosome uptake did not differ in response to androgen, a higher exosomal miR-379-5p content of DHT-exo (exosomes derived from DHT-treated granulosa cells), suppressed androgen-induced proliferation by increasing its cellular content in preantral follicle granulosa cells. In breast cancer cells, miR-379 has been reported to be a tumor suppressor, and its expression is significantly lower in cancer compared with healthy breast tissues [59]. Recently, mesenchymal stem cells stably transduced to express miR-379 and exosome-enriched with miR-379-5p have been developed for breast cancer therapy. Systemic administration of exosome enriched with miR-379 has been shown to have therapeutic effect, with a significant reduction in tumor activity in animals [59]. These results support the regulatory role of exosomes in the determination of cellular miR-379-5p content and proliferation.

Recently, we have demonstrated that one-month of DHT treatment reduces ovarian weight and length, but without affecting pre-antral follicles atresia and systemic changes, including body weight and the insulin sensitivity index [14]. Although all stage of follicles and corpus luteum can be found in control ovaries, DHT ovaries mainly contain pre- and early antral follicles. In the current study, overexpression of miR-379-5p in ovaries using lentiviral injection allowed us to further evaluate its function in follicular development. MiR-379-5p injection suppressed preantral follicle granulosa cell proliferation and development without affecting other stages of follicular development in both control and androgenized model, suggesting that miR-379-5p is mainly involved in the development of preantral follicles.

MiR-379-5p content in follicular fluid from PCOS subjects was significantly reduced in exosomes with no changes in extracellular vesicle-depleted follicular fluid, whereas androgen-induced miR-379-5p changes in rat granulosa cells was exosome-specific and was not detectable in extracellular vesicle-depleted condition medium.

Although, androgen did not influence exosomal miR-379-5p content in antral follicles stage in rat, human PCOS significantly had lower exosomal miR-379-5p content. This is important to consider that human granulosa cells and follicular fluids were collected at the preovulatory follicle stage; however those of rat collected from antral follicles. Therefore, the differences of exosomal miR-379-5p content could have be resulted from differences in follicular stage between the rat and the human models.

In conclusion, our findings suggest that increased exosomal miR-379-5p release from granulosa cells in response to androgen action is a proliferative mechanism specific to preantral follicle development. To facilitate future investigation into the role and regulation of miR-379-5p in the androgenic control of ovarian follicular development, the following hypothetical model is proposed (Supplementary Fig. 8). In preantral follicles, androgen excess reduces granulosa cell miR-379-5p content by increased exosomal release. Reduced cellular miR-379-5p up-regulates PDK1, which phosphorylates and activates AKT, and induces cell proliferation. In contrast, these cellular mechanisms were not evident at the antral follicle stage, resulting in attenuation of the proliferative response of granulosa cells to androgenic stimulation and of antral follicle growth. Although it is well established that exosomes facilitate miRNA-mediated cell–cell communication, whether and how target cell uptake of exosomes enriched in miR-379-5p within the ovarian microenvironment regulates follicular growth in normal ovarian physiology, and in the etiology of PCOS, is unknown and requires further investigation.

## Materials and methods

### Reagents and antibodies

Cell culture media (M199), fetal bovine serum (FBS), penicillin and streptomycin, L-glutamine, sodium pyruvate, and trypsin were purchased from Invitrogen (Burlington, Canada). Diethylstilbestrol (DES), equine chorionic gonadotropin (eCG), HEPES, bovine serum albumin (BSA), bovine insulin, transferrin, ascorbic acid, and sodium selenite anhydrous were from Sigma (St. Louis, MO). 5α-dihydrotestosterone (DHT) was obtained from Steraloids (Newport, RI). Anti-rabbit and -mouse IgG conjugated with horseradish peroxidase and reagents for SDS-PAGE were purchased from Bio-Rad Laboratories (Mississauga, Ontario, Canada). Enhanced chemiluminescent reagent was from Thermo Fisher Scientific (Rockford, IL).

### Animals and DHT implant in vivo

Female Sprague Dawley rats (Charles River, Montreal, Canada) were maintained on 12 h cycle (light and dark) and given food and water ad libitum. Immature female rats at 21 days of age were implanted subcutaneously with silicone capsules without (control, sham control) or with DHT (DHT, Steraloids Inc., Newport, USA), as previously described [12, 15] to continuously release 83 μg DHT/day for 28 days. Sham control animals received identical pellets lacking the steroid. As ovaries of androgenized rats contain mainly preantral follicles, we only isolated preantral follicle granulosa cells from control and DHT-treated rats in this experiment. Granulosa cells (*n* = 3 replicates, each from 1 rat) from both control and DHT-treated rats were isolated by follicular puncture and were kept frozen in -80 °C until analysis.

### Primary culture of rat follicles, granulosa cells, miRNA transfection and exosome release inhibition

Granulosa cells from preantral follicles (Diethylstilbestrol [DES]-primed 21-day old rats; 1 mg/d, subcutaneous injection [s.c.]for 3 consecutive days) and antral follicles (Equine chorionic gonadotropin [eCG]–injected 22-day old rats; 10 IU, intraperitoneal injection [i.p.], animals sacrificed 2 days post-injection) were isolated by follicular puncture as described previously [9, 15].

Granulosa cells were plated (1 × 10^6^ per well in a 6 well plate) overnight in M199 with 10% FBS under a humidified atmosphere of 95% air and 5% CO2. After culture overnight in serum-free medium, granulosa cells were treated with or without DHT (1 µM; 24 h and 36 h; (*n* = 3 replicates, each from 2 rats)). DMSO and alcohol were added to the control group (final concentration of 0.001% and 0.005%, respectively) as they were used as a vehicle for DHT. Our laboratory has previously examined different concentrations of DHT on various ovarian parameters in vitro [10], including follicular growth, granulosa cell proliferation, apoptosis, steroidogenesis, androgen receptor content. We found that 1 μM is the optimal concentration, and this concentration was used in the current study. In some experiments, granulosa cell (*n* = 3 replicates, each from 2 rats) cultures were transfected with miR-379-5p mimic (50 and 100 nM) or inhibitor (100 nM; mirVana Mimics & Inhibitors, Life Technologies, Inc.) or scrambled sequence using Lipofectamine 2000 (Life Technologies, Inc.) for 24 h before DHT treatment in vitro [47]. To inhibit exosome release, granulosa cells (*n* = 3 replicates, each from 2 rats) were treated with GW4869 (0, 10 and 20 mM, Cayman Chemical, Ann Arbor, MI) for 24 h before DHT treatment [47].

Preantral follicles (110 to 150 mm diameter) were mechanically isolated from 13- to 14-day-old rats, individually cultured in Leibowitz L-15 medium with BSA (0.1%, wt/vol; 96 well dish) and their growth assessed as described previously [9, 15]. Briefly, only round follicles with intact basement membrane and theca layer were selected for the present studies. Follicles were cultured individually for 4 d in a 96-well plate in 100 μl of α-MEM supplemented with HEPES (10 mM), BSA (0.1%, wt/vol), bovine insulin (5 μg/ml), transferrin (2 μg/ml), ascorbic acid (25 μg/ml), sodium selenite anhydrous (2 ng/ml), L-glutamine (3 mM), sodium pyruvate (100 μg/ml), streptomycin (100 μg/ml), penicillin (100 U/ml), and FSH (10 ng/ml). The culture medium was changed every other day. Follicular diameter was measured daily as the average distance between the outer edges of the basement membrane in two perpendicular planes, and results were expressed as change in follicular volume [9, 15].

Exosome release inhibition and miRNA transfection in preantral follicles were carried out by using *n* = 15 follicles in each experimental group as described above. Alexa-647-labelled miR-379-5p mimic was used to confirm successful transfection of granulosa cells in preantral follicles. Transfected preantral follicles were imaged by Zeiss LSM880 with AiryScan FAST at the Cell Biology and Image Acquisition of the University of Ottawa. Control group received unlabeled scrambled sequence using Lipofectamine 2000 (Life Technologies, Inc.).

### Human samples

Human ovarian follicular fluids (FF) were collected from pre-ovulatory follicle of lean PCOS (based on Rotterdam criteria; BMI < 30; Supplementary table 2) and non-PCOS subjects (premenopausal, 18–40 years) with normal thyroid function and prolactin levels, undergoing assisted reproduction treatment at the CReATe Fertility Centre, Toronto, Canada. Exclusion criteria include: (1) known causes of oligomenorrhea other than PCOS; and (2) use of hormone treatment, birth control pill, insulin sensitizers, lipid lowering agents, or medications known to influence insulin sensitivity or serum androgens, within 3 months of the study onset. Moreover, follicular fluids were collected individually from pre-ovulatory follicle of right or left ovary without buffer and those contaminated with visible blood were excluded (Supplementary Fig. 9). All FF (PCOS, *n* = 13 & non-PCOS, *n* = 25) and granulosa cell (Ki67: pre-ovulatory follicle; PCOS, *n* = 12 & non-PCOS, *n* = 11) specimens were obtained from the CReATe Biobank, CReATe Fertility Centre [60].

### Adenovirus infection

Granulosa cells were infected with adenoviral sense full-length cortactin cDNA or control adenoviral vector at 50 MOI, as previously described [10]; and then treated with or without DHT (1 µM; 24 h and 36 h; *n* = 3 replicates, each from 2 rats).

### Protein extraction and western blotting

Granulosa cell cultures were harvested by trypsin treatment and lysed using Cell Lysis Buffer (Cat#: 9803; Cell Signaling Technology, Inc; Beverly, Massachusetts). Protein extraction and western blotting were performed as described previously [15]. Antibodies and their dilutions are summarized in the Supplementary table 1.

### Exosome and microvesicle isolation and nanoparticle tracking analysis

Extracellular vesicles in granulosa cell conditioned medium and human follicular fluids were isolated by differential centrifugation [47] [Apoptotic bodies: 2500 × g, 10 min, 4˚C; Microvesicles: 20,000 × g, 20 min, 4˚C; Exosomes: granulosa cell conditioned medium (100,000 × g for 90 min at 4˚C) and human follicular fluids (ExoQuick; System Biosciences [61])]. Extracellular vesicles were characterized by nanoparticle tracking analysis (Zetaview) and western blotting (CD63) [47].

Extracellular vesicles in phosphate-buffered saline (PBS) were analyzed for size and number by using the ZetaView PMX110 Multiple Parameter Particle Tracking Analyzer (Particle Metrix, Meerbusch, Germany), and ZetaView software version 8.02.28 as previously described [62, 63]. Extracellular vesicles were captured by 11 camera positions at 21 °C, and the pellet size and concentration was evaluated.

### miRNA isolation, real-time PCR, luciferase assay

Total RNA, inclusive of the small RNA fraction, was extracted using the miRNeasy Mini Kit (Qiagen Inc., Toronto, ON, Canada). The TaqMan MicroRNA Reverse Transcription Kit (Life Technologies, Inc.) and TaqMan Advanced miRNA cDNA Synthesis Kit (Life Technologies, Inc.) were used for cDNA preparation, as per the manufacturer’s instructions. Real-time PCR reactions were performed by TaqMan Advanced miRNA Assays and Taqman miRNA assay from Thermo Fisher (Life Technologies, Inc.) on human and rat samples, respectively in an Applied Biosystems 7500 sequence detection real-time PCR system (Foster City, CA) (See Supplementary Table S3). Since miR-92a-3p level in human follicular fluids are not influenced by PCOS (based on miRNA profiling results from Next Generation Sequencing; Wyse et al., Unpublished). miR-92a-3p and U6 RNA were used to normalize miR-379-5p content in human and rat samples, respectively. The relative amount of miR-379-5p to U6 and miR-92a-3p RNA was expressed using the 2-ΔΔCt method [64].

Transfection studies were conducted using firefly luciferase as a reporter gene and normalized using Renilla Luciferase (GeneCopoeia, Rockville, MD) [47]. The pEZX- MT06-PDK1 3ʹUTR-f/rLuc or mutant PDK1 3ʹUTR vectors (100 ng, GeneCopoeia) were transfected into preantral granulosa cells with miR-379-5p mimic or scrambled sequence (100 nM; *n* = 3 replicates, each from 2 rats; mirVana Mimics & Inhibitors, Life Technologies, Inc.). After 24 h, luciferase activity was assayed using a Dual Luminescence assay kit (GeneCopoeia) and read on an Orion II microplate luminometer (Berthold Detection Systems, Pforzheim, Germany).

### Exosomal uptake analysis

Preantral granulosa cells (donor cells) were cultured (60 mm dishes at 2 × 10^6^ cells/ dish) without (CTR-exo) or with DHT (DHT-exo) and exosomes were isolated from conditioned medium after 36 h. To prepare recipient cells, preantral follicle granulosa cells were plated (M-199 containing 10% FBS, 18 h, 12-well dishes at 3 × 10^5^ cells per well; *n* = 3 replicates) and pre-treated with or without DHT for 12 h (M-199, 18 h). Exosomes were isolated by using ultracentrifugation, re-suspended in PBS and incubated with recipient granulosa cells (10 μg protein/100,000 granulosa cells for 24 h; Bradford protein assay [15, 65]. Proliferative response (CCK-8 assay; Sigma) and exosome uptake were assessed in the following 4 experimental groups: (1) CTR recipient cells + CTR-exo, (2) DHT recipient cells + CTR-exo, (3) CTR recipient cells + DHT-exo, and (4) DHT recipient cells + DHT-exo.

To evaluate exosome uptake, exosomes from donor cells were labeled with the red fluorescent dye PKH26 (Sigma) for 5 min at room temperature and then incubated with recipient granulosa cells (10 μg protein/100,000 granulosa cells) for 24 h [47, 66, 67]. Granulosa cells were washed with PBS once before detaching by cell scraper (Corning™ Falcon™). Granulosa cells (10^5^ cells) were stained with viability dye eFluor 450 in PBS (Biolegend, San Diego, USA; 30 min, 4◦C), washed with FACS buffer (PBS with 1% BSA), and re-suspended in 1% of paraformaldehyde. To compare exosome uptake among treatments, flow cytometric acquisition was performed and analyzed as described previously [12].

### Production of recombinant lentiviral particles, injection and immunofluorescence

The lentiviral gene transfer plasmids pLV-[hsa-miR-379-5p] (Cat.no. miR-p209m) and pLV-[miR-control] (Cat.no. mir-p000) were purchased from the BioSettia Company (San Diego, California, USA); production of recombinant lentiviral particles was performed as per manufacturer’s protocol. Briefly, HEK 293 T cells were co-transfected with the transfer vector and the helper plasmids pMD2.G (Addgene) and psPAX2 (Addgene), using the calcium phosphate co-precipitation method. Prior to transfection, a total of 6 × 10^6^ 293 T cells were seeded in 10 cm plates for 24 h in modified Dulbecco’s culture medium containing FBS (10%), penicillin (100 IU/ml), and streptomycin (100 mg/ml) in 5% CO_2_. The culture medium was changed one hour prior to transfection and a total of 18 μg of plasmid DNA was added per dish: [envelope plasmid pMD2.G (3 μg), packaging plasmid psPAX2 (6 μg) and transfer vector plasmid (9 μg)]. The precipitate, formed from adding the plasmids to a final volume of 540 μl and 60 μl of 2.5 M CaCl2 and then dropwise 600 ml of 2 × HEPES-buffered saline, was added immediately to the cultures. The medium was then replaced every 24 h with fresh medium for high-concentration virus production. The high-titered virus was achieved through serial ultracentrifugation, as previously described [68]. Briefly, the viral supernatant was collected and filtered through a 0.45 µm filter and transferred into sterilized Ultra-Clear centrifuge tubes (Beckman cat. no. 344058). The viral supernatant was centrifuged (16,500 × g, 90 min, 4°C), pooled, and stored in aliquots at -80°C.

Animals were randomly divided into 2 groups (control and DHT implants) and left and right ovaries in each animal were injected with RFP virus particles and miR-379-5p mimic, respectively, sacrificed 28 days post-injection, and ovarian growth (weight and length), number of follicles at each stage, and their cell proliferation (Ki67) at each stage, were compared. Viral particles containing RFP or miR-379-5p were injected using a syringe with a glass micro-injection needle (50 μm diameter), as described previously [69]. Effective transduction of the granulosa cells with lentivirus particles (2 µl) was confirmed seven days post-injection by assessing the RFP signal in ovarian sections with the IVIS Spectrum system (PerkinElmer). Additionally, RFP mRNA expression in the granulosa cells was also assessed. Our results indicate that all four experimental groups expressed RFP mRNA (Supplementary Fig. 10), supporting the notion that granulosa cells were successfully transduced by lentivirus.

To confirm the lentivirus transduction efficiency of granulosa cells, we assessed the mRNA expression of red fluorescent protein (RFP; Forward: cccgtaatgcagaagaaga; Reverse: ggccttgtaggtggtcttga; Supplementary Fig. 10) in granulosa cells isolated from control and lentivirus treated rats. Total RNA was extracted with Trizol and reverse transcribed into cDNA using a High-Capacity cDNA Reverse Transcription Kit (Applied Biosystems), as per the manufacturer’s instructions. Real-time PCR reactions were performed by Fast SYBR Green Master Mix (Applied Biosystems) in an Applied Biosystems 7500 sequence detection real-time PCR system (Foster City, CA) [70]. Melting curve analyses were performed at the end of each run to ensure specificity of the amplification. RFP primer efficiency was determined with a standard curve (with at least five serial dilutions) in a pool of all samples. Data were analyzed by the 2-ΔΔCT method [64].

At the time of animal sacrifice, the ovaries were sampled and trimmed according to guidelines for organ sampling in rats and mice published by RITA (Registry of Industrial Toxicology Animal-data) and NACAD (North American Control Animal Database) group (Kittel B. et al. Revised guides for organ sampling and trimming in rats and mice – Part 2. Exp Toxic Pathol 2004; 55: 413–431). Ovaries were fixed in 4% formalin, dehydrated, and embedded in paraffin. After trimming off 400–500 μm of the tissue, 4 µm longitudinal sections were made from the central part of each ovary. Since rat follicles are relatively big structures (around 100–300 μm), a middle section represents about 60–70% of all follicles in the ovary. Therefore, the number of preantral, early antral, antral, preovulatory follicles, and corpus luteum was counted on a middle section. The ovarian histopathological assessment and follicle counting were performed with hematoxylin and eosin (H&E) staining [71] on whole slide images based on Goldman et al. (PNAS 2017; PMID: 28,270,607) and our previous publication [47, 66, 67]. For immunofluorescence studies, sections were deparaffinized in xylene and rehydrated through a 100–70% ethanol gradient. Antigen retrieval was achieved by boiling slides in TRIS/EDTA (pH 9.0) or citrate (pH 6.0) buffer in a microwave for 15 min. Sections were blocked with 10% donkey serum in 1% BSA in phosphate-buffered saline (40 min) before incubation with rabbit anti-Ki67 (overnight, 4°C, Abcam, ab16667; 1:200). Donkey anti-rabbit Cy3 as secondary antibody (Jackson Immunoresearch, AB-2307443; 1:100) was incubated for 1 h at room temperature. The fluorescent dye Hoechst 33,342 (1:10,000; Molecular Probes) was used for nuclei detection. Sections were protected with VectaShield mounting medium (Vector Labs), scanned by Axio Scan.Z1 (Carl Zeiss, Gottingen, Germany) and recorded with the Axion Vision program (Axion Vision software, Zeiss). The macro (FIJI software) was used to apply colour threshold based selection for the respective signals/channels which then counted the selected particles. 

### Human granulosa cell proliferation (Ki67)

Human granulosa cells (2 × 10^5^ cells) were fixed, permeabilized, stained for Ki67 and flow cytometric acquisition was performed and analyzed as described previously [12].

### Statistics

T-tests, and one-, two-, or three-way analyses of variance (ANOVA) were used to assess the effects of, and interactions between variables, followed by multiple comparison by the Tukey post hoc test, using Prism v.7 (GraphPad, San Diego, CA) and Sigma plot v.12 (Systat Software, San Jose, CA). *P* < 0.05 was considered statistically significant.

### Study approval

All FF and granulosa cell specimens were obtained from the CReATe Biobank, CReATe Fertility Centre. The CReATe Biobank (banking protocols approved by Veritas IRB), collects biological materials from consenting patients, according to the best practice-based standards of biobanking [60]. All samples from the Biobank were approved for use in this study by the University of Toronto REB (Protocol#29,236) and The Ottawa Hospital REB (Protocol #20,170,453-01H)]. All animal procedures were carried out in accordance with the Guidelines for the Care and Use of Laboratory Animals, Canadian Council on Animal Care, and were approved by the University of Ottawa Animal Care Committee.

## Supplementary Information

**Additional file 1:** **Supplementary Figure 1. **Androgen excess in human PCOS subjects is associated with reduced follicular fluid-derived exosomal mir-379-5p content and granulosa cell proliferation. (A) Follicular fluids from the dominant follicles (≥ 20 mm; *n* = 25 Non-PCOS and 13 PCOS subjects) of PCOS subjects exhibited significantly higher free testosterone level and lower mir-379-5p contents (relative to mir-92a-3p, determined by Next Generation sequencing) in exosomes. mir-379-5p was detected in extracellular vesicle (EV)-depleted follicular fluid (FF) but its levels was not different between PCOS and Non-PCOS subjects. (B & C) Granulosa cells from PCOS subjects had significantly lower proliferation (*n* = 12 Non-PCOS and 11 PCOS subjects) than those of non-PCOS subjects. MiRNA expression was assessed by TaqMan Advanced miRNA Assays (Thermo Fisher). Results are expressed as means ± SEM. Data were analyzed by t-test and Pearson correlation. *P < 0.05, **P < 0.01 and ****P < 0.0001. **Supplementary figure 2.** Androgen does not influence cellular and exosomal contents of mir-24, mir-9 and let-7d in rat pre-antral follicle granulosa cells.  Granulosa cells were isolated from preantral follicles (Diethylstilbestrol-primed 21-day old rats; 1 mg/d, subcutaneous injection for 3 consecutive days). Granulosa cells were cultured with DHT (0 and 1 µM, 24 h and 36 h). Exosomes were isolated from granulosa cell-conditioned medium by differential centrifugation and their size and concentrations were determined by nanoparticle tracking analysis. miRNA expression was assessed by TaqMan miRNA Assays (Thermo Fisher) and normalized to U6. Results are expressed as means ± SEM (*n* = 3 replicates, each from 2 rats). Data were analyzed by two-way ANOVA and tukey post hoc. **Supplementary figure 3**. DHT treatment did not affect granulosa cell TGFBR1 protein content in rat preantral and antral follicles *in vitro*. Granulosa cells were isolated from preantral follicles (Diethylstilbestrol-primed 21-day old rats; 1 mg/d, subcutaneous injection for 3 consecutive days) and antral follicles (Equine chorionic gonadotropin –injected 22-day old rats; 10 IU intraperitoneal injection; animal sacrificed 48 h post-treatment). Granulosa cells were cultured with DHT (0 and 1 µM) for 24 h and 36 h. **Supplementary figure 4.** Androgen increased the cellular content of pri-miR-379 in rat granulosa cells. Granulosa cells were isolated from preantral follicles (Diethylstilbestrol-primed 21-day old rats; 1 mg/d, subcutaneous injection for 3 consecutive days) and antral follicles (Equine chorionic gonadotropin –injected 22-day old rats; 10 IU intraperitoneal injection; animal sacrificed 48 h post-treatment). Granulosa cells were cultured with DHT (0 and 1 µM) for 24 h and 36 h. miRNA expression was assessed by TaqMan miRNA Assays (Thermo Fisher) and normalized to 𝛽-actin. Results are expressed as means ± SEM (*n* = 3 replicates, each from 2 rats). Data were analyzed by two-way ANOVA and tukey post hoc. **Supplementary figure 5.** The median size of exosomes and microvesicles isolated from rat pre-antral and antral follicle granulosa cell-conditioned medium. Granulosa cells were isolated from preantral follicles (Diethylstilbestrol-primed 21-day old rats; 1 mg/d, subcutaneous injection for 3 consecutive days) and antral follicles (Equine chorionic gonadotropin –injected 22-day old rats; 10 IU intraperitoneal injection; animal sacrificed 48 h post-treatment). Granulosa cells were cultured with DHT (0 and 1 µM) for 24 h and 36 h. Exosomes and microvesicles were isolated from granulosacell-conditioned medium by differential centrifugation as described in Materials and Methods. Results are expressed as means ± SEM (*n* = 3 replicates, each from 2 rats). Data were analyzed by three-way ANOVA and tukey post hoc. **Supplementary figure 6.** Exosome release is inhibited by GW4869 at 20 nM. (A) CD63 protein is an exosome marker and is expressed only in exosome fraction of granulosa cell conditioned medium. (B) GW4869 (20 nM) effectively suppressed exosome release, as evident by reduced CD63 protein content in conditioned medium. Granulosa cells were isolated from preantral follicles (Diethylstilbestrol-primed 21-day old rats; 1 mg/d, subcutaneous injection for 3 consecutive days). Exosomes were isolated from granulosa cell-conditioned medium by differential centrifugation as described in Materials and Methods. **Supplementary Figure 7**. Reconstitution of DHT-treated (mir-379-5p down-regulated) rat granulosa cells with mir-379-5p-enriched exosomes attenuates the DHT-induced proliferative response *in vitro*. (A) Experimental design: Preantral granulosa cells (donor cells) were treated without (CTR-exo) or with DHT (DHT-exo), and exosome were isolated from spent culture medium 36 h post-treatment. To prepare recipient cells, granulosa cells from preantral follicles were pre-treated without or with DHT for 12 h. Exosomes were labeled with the red fluorescent dye PKH26 by incubating with granulosa cell (10 μg protein/100,000 granulosa cells) for 24 h. Granulosa cells (105 cells) were stained with viability dye eFluor 450. (B) Exosomal uptake was assessed in viable granulosa cells by flow cytometry in the following 4 experimental groups: (1) CTR recipient cells + CTR-exo, (2) DHT recipient cells + CTR-exo, (3) CTR recipient cells + DHT-exo, and (4) DHT recipient cells + DHT-exo; (C) While androgen excess failed to alter exosomal uptake,  culture of DHT-treated granulosa cells (mir-379-5p down-regulated) with mir-379-5p-enriched exosomes (DHT-exo) attenuates the DHT-induced proliferative response *in vitro*. Granulosa cells were isolated from preantral follicles (Diethylstilbestrol-primed 21-day old rats; 1 mg/d, subcutaneous injection for 3 consecutive days). Results are expressed as means ± SEM (*n* = 3 replicates). Data were analyzed by two-way ANOVA and tukey post hoc. *P < 0.05. **Supplementary Figure 8.** A hypothetical model illustrating androgen-induced exosomal mir-379-5p release from granulosa cells removes its inhibitory action on PDK1, a proliferative mechanism specific for preantral follicle granulosa cells. Androgen excess reduces granulosa cell mir-379-5p content by increasing its exosomal release in preantral follicles, but not in antral follicles. Reduced granulosa cell mir-379-5p content increases PDK1-mediated cell proliferation. Consequently, androgen excess promotes preantral but suppresses antral follicular development, as observed in PCOS. (Created in BioRender.com). **Supplementary figure 9.** Guide for inclusion or rejection of follicular fluid samples for study. **Supplementary figure 10.** Validation of lentivirus transduction following intrabursal injection. (A) Red fluorescent protein (RFP) signal in rat ovarian sections was detectable 7 days following intrabursal injection of lentivirus-RFP and saline (control), demonstrating successful delivery and transduction; (B) RFP mRNA expression was detectable (normalized to β-actin) in granulosa cells isolated from rats injected with lentivirus. Granulosa cells isolated from untreated rats were considered as Control. Results are expressed as means ± SEM; *n* = 3 per group, each from 2 rats. Data were analyzed by one-way ANOVA and tukey post hoc. **P < 0.01, ***P < 0.001. **Supplementary Table 1.** List of antibodiesand their dilution used in this study. **Supplementary Table 2.** Patient characteristics. **Supplementary Table 3.** List of TaqMan Advanced miRNA Assays and Taqman miRNA assay (Life Technologies, Inc.) used in this study.
