# Supplementary material for: Androgen-induced exosomal miR-379-5p release determines granulosa cell fate: cellular mechanism involved in polycystic ovaries
Source: J Ovarian Res. 2023 Apr 12;16:74. doi: 10.1186/s13048-023-01141-1 (PMC10091561; doi:10.1186/s13048-023-01141-1)
Supplement: Supplementary file 1 — Additional file 1: Supplementary Figure 1. Androgen excess in human PCOS subjects is associated with reduced follicular fluid-derived exosomal mir-379-5p content and granulosa cell proliferation. (A) Follicular fluids from the dominant follicles (≥ 20 mm; n = 25 Non-PCOS and 13 PCOS subjects) of PCOS subjects exhibited significantly higher free testosterone level and lower mir-379-5p contents (relative to mir-92a-3p, determined by Next Generation sequencing) in exosomes. mir-379-5p was detected in extracellular vesicle (EV)-depleted follicular fluid (FF) but its levels was not different between PCOS and Non-PCOS subjects. (B & C) Granulosa cells from PCOS subjects had significantly lower proliferation (n = 12 Non-PCOS and 11 PCOS subjects) than those of non-PCOS subjects. MiRNA expression was assessed by TaqMan Advanced miRNA Assays (Thermo Fisher). Results are expressed as means ± SEM. Data were analyzed by t-test and Pearson correlation. *P < 0.05, **P < 0.01 and ****P < 0.0001. Supplementary figure 2. Androgen does not influence cellular and exosomal contents of mir-24, mir-9 and let-7d in rat pre-antral follicle granulosa cells. Granulosa cells were isolated from preantral follicles (Diethylstilbestrol-primed 21-day old rats; 1 mg/d, subcutaneous injection for 3 consecutive days). Granulosa cells were cultured with DHT (0 and 1 µM, 24 h and 36 h). Exosomes were isolated from granulosa cell-conditioned medium by differential centrifugation and their size and concentrations were determined by nanoparticle tracking analysis. miRNA expression was assessed by TaqMan miRNA Assays (Thermo Fisher) and normalized to U6. Results are expressed as means ± SEM (n = 3 replicates, each from 2 rats). Data were analyzed by two-way ANOVA and tukey post hoc. Supplementary figure 3. DHT treatment did not affect granulosa cell TGFBR1 protein content in rat preantral and antral follicles in vitro. Granulosa cells were isolated from preantral follicles (Diethylstilbestrol-primed 21-da [file 13048_2023_1141_MOESM1_ESM.pptx]

## Slide 1
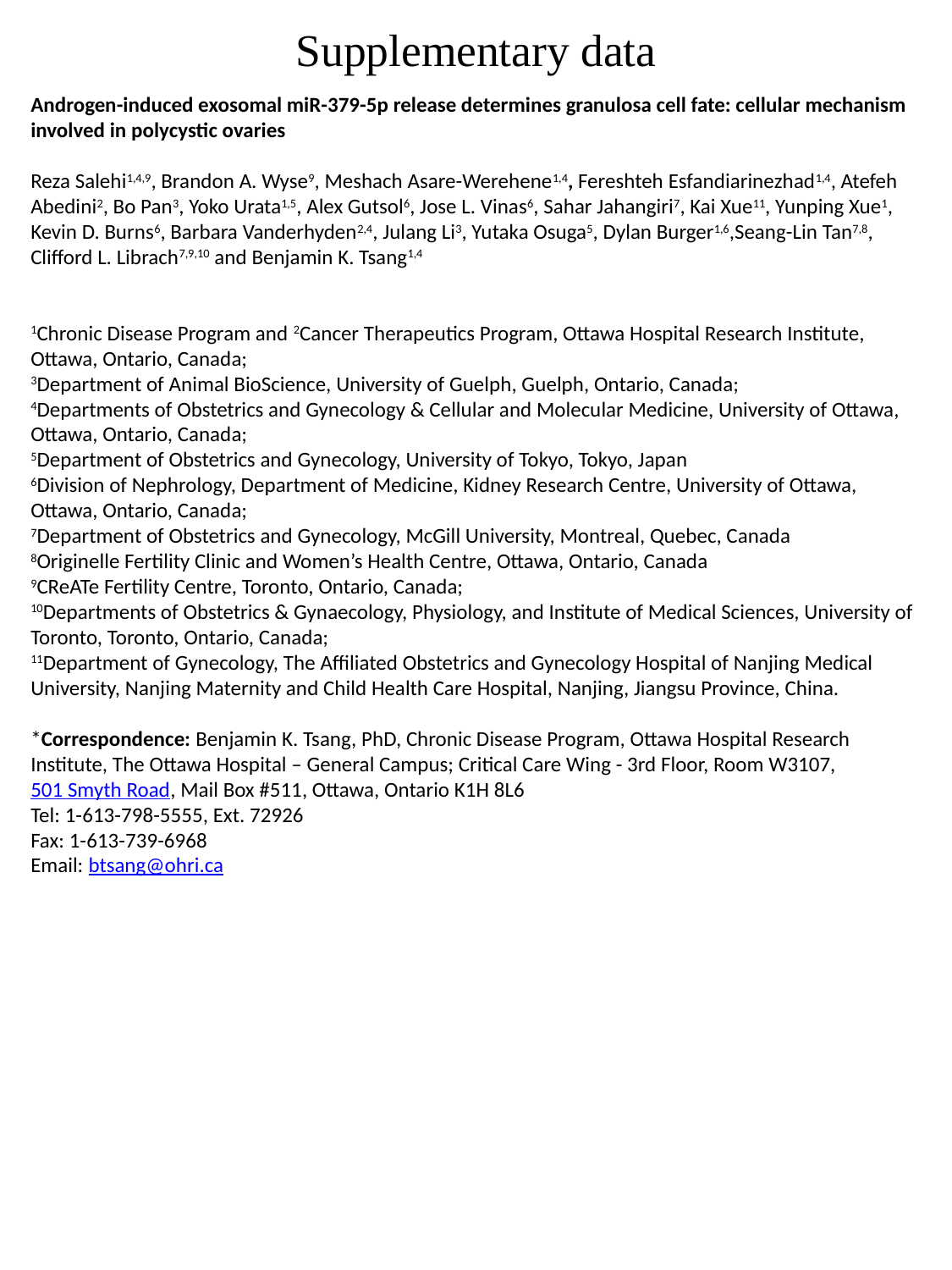

# Supplementary data
Androgen-induced exosomal miR-379-5p release determines granulosa cell fate: cellular mechanism involved in polycystic ovaries
Reza Salehi1,4,9, Brandon A. Wyse9, Meshach Asare-Werehene1,4, Fereshteh Esfandiarinezhad1,4, Atefeh Abedini2, Bo Pan3, Yoko Urata1,5, Alex Gutsol6, Jose L. Vinas6, Sahar Jahangiri7, Kai Xue11, Yunping Xue1, Kevin D. Burns6, Barbara Vanderhyden2,4, Julang Li3, Yutaka Osuga5, Dylan Burger1,6,Seang-Lin Tan7,8, Clifford L. Librach7,9,10 and Benjamin K. Tsang1,4
1Chronic Disease Program and 2Cancer Therapeutics Program, Ottawa Hospital Research Institute, Ottawa, Ontario, Canada;
3Department of Animal BioScience, University of Guelph, Guelph, Ontario, Canada;
4Departments of Obstetrics and Gynecology & Cellular and Molecular Medicine, University of Ottawa, Ottawa, Ontario, Canada;
5Department of Obstetrics and Gynecology, University of Tokyo, Tokyo, Japan
6Division of Nephrology, Department of Medicine, Kidney Research Centre, University of Ottawa, Ottawa, Ontario, Canada;
7Department of Obstetrics and Gynecology, McGill University, Montreal, Quebec, Canada
8Originelle Fertility Clinic and Women’s Health Centre, Ottawa, Ontario, Canada
9CReATe Fertility Centre, Toronto, Ontario, Canada;
10Departments of Obstetrics & Gynaecology, Physiology, and Institute of Medical Sciences, University of Toronto, Toronto, Ontario, Canada;
11Department of Gynecology, The Affiliated Obstetrics and Gynecology Hospital of Nanjing Medical University, Nanjing Maternity and Child Health Care Hospital, Nanjing, Jiangsu Province, China.
*Correspondence: Benjamin K. Tsang, PhD, Chronic Disease Program, Ottawa Hospital Research Institute, The Ottawa Hospital – General Campus; Critical Care Wing - 3rd Floor, Room W3107, 501 Smyth Road, Mail Box #511, Ottawa, Ontario K1H 8L6
Tel: 1-613-798-5555, Ext. 72926
Fax: 1-613-739-6968
Email: btsang@ohri.ca

## Slide 2
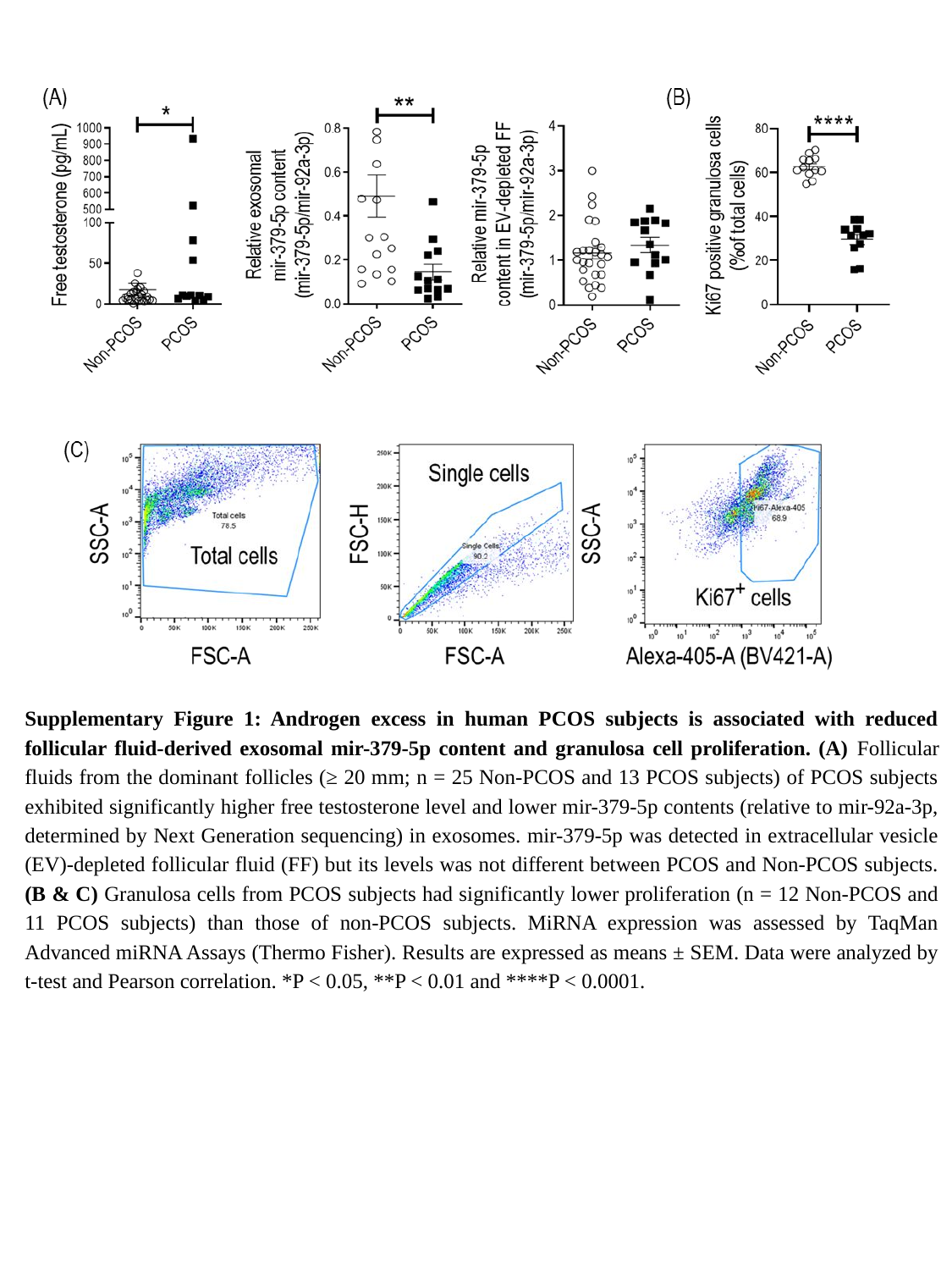

Supplementary Figure 1: Androgen excess in human PCOS subjects is associated with reduced follicular fluid-derived exosomal mir-379-5p content and granulosa cell proliferation. (A) Follicular fluids from the dominant follicles (≥ 20 mm; n = 25 Non-PCOS and 13 PCOS subjects) of PCOS subjects exhibited significantly higher free testosterone level and lower mir-379-5p contents (relative to mir-92a-3p, determined by Next Generation sequencing) in exosomes. mir-379-5p was detected in extracellular vesicle (EV)-depleted follicular fluid (FF) but its levels was not different between PCOS and Non-PCOS subjects. (B & C) Granulosa cells from PCOS subjects had significantly lower proliferation (n = 12 Non-PCOS and 11 PCOS subjects) than those of non-PCOS subjects. MiRNA expression was assessed by TaqMan Advanced miRNA Assays (Thermo Fisher). Results are expressed as means ± SEM. Data were analyzed by t-test and Pearson correlation. *P < 0.05, **P < 0.01 and ****P < 0.0001.

## Slide 3
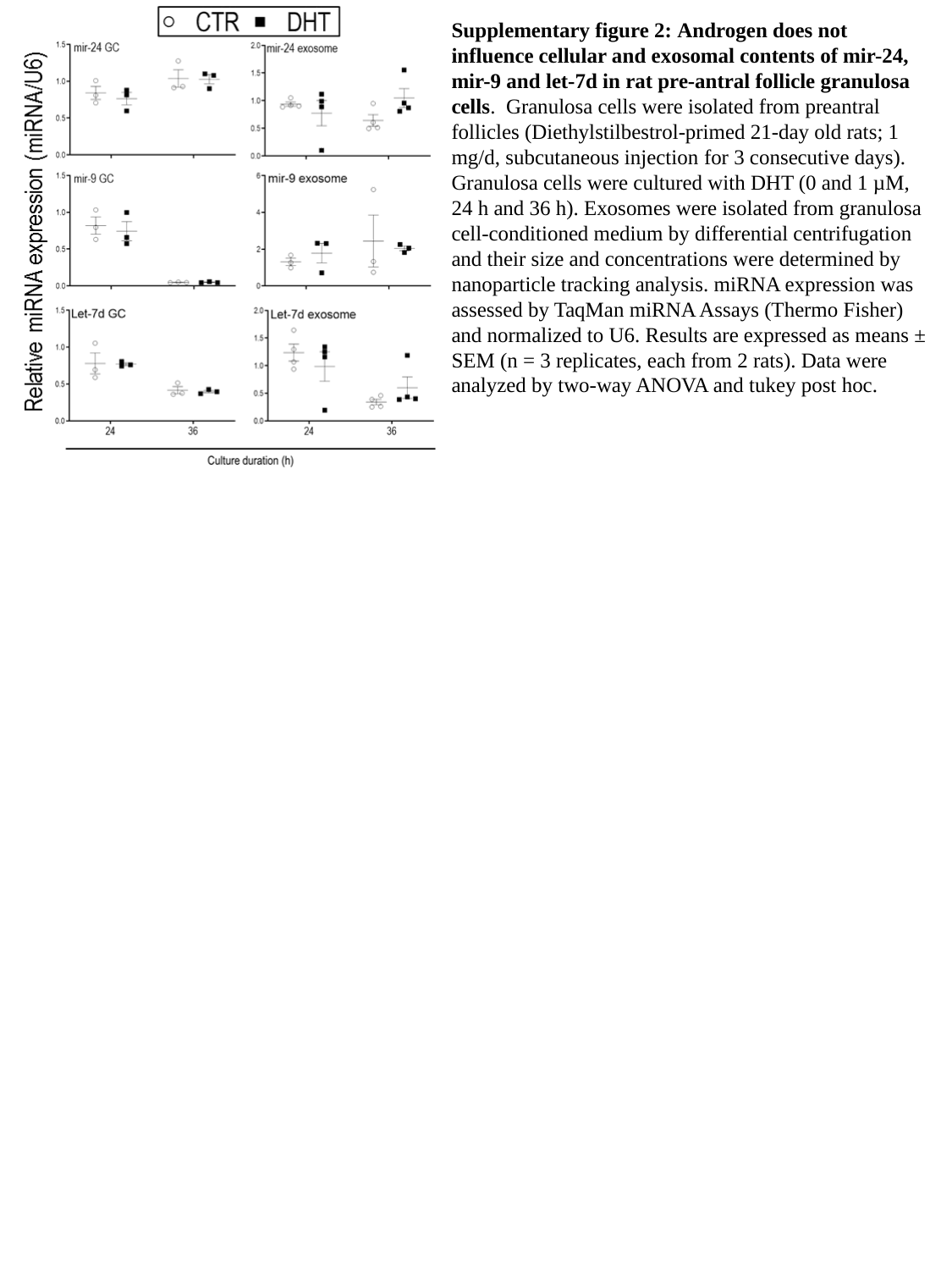

Supplementary figure 2: Androgen does not influence cellular and exosomal contents of mir-24, mir-9 and let-7d in rat pre-antral follicle granulosa cells. Granulosa cells were isolated from preantral follicles (Diethylstilbestrol-primed 21-day old rats; 1 mg/d, subcutaneous injection for 3 consecutive days). Granulosa cells were cultured with DHT (0 and 1 µM, 24 h and 36 h). Exosomes were isolated from granulosa cell-conditioned medium by differential centrifugation and their size and concentrations were determined by nanoparticle tracking analysis. miRNA expression was assessed by TaqMan miRNA Assays (Thermo Fisher) and normalized to U6. Results are expressed as means ± SEM (n = 3 replicates, each from 2 rats). Data were analyzed by two-way ANOVA and tukey post hoc.

## Slide 4
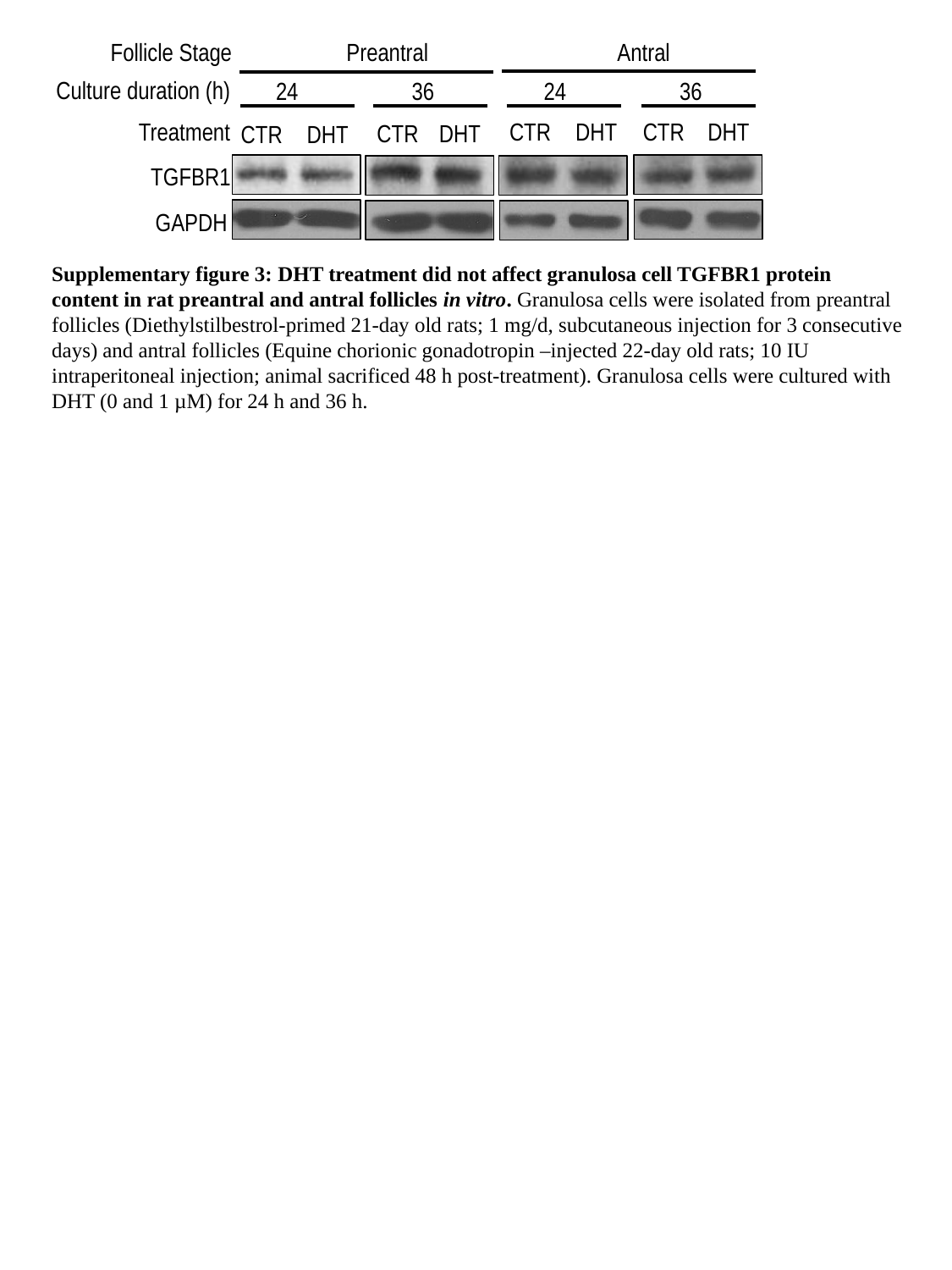

Follicle Stage
Preantral
Antral
Culture duration (h)
24
36
24
36
Treatment
CTR
DHT
CTR
DHT
CTR
DHT
CTR
DHT
TGFBR1
GAPDH
Supplementary figure 3: DHT treatment did not affect granulosa cell TGFBR1 protein content in rat preantral and antral follicles in vitro. Granulosa cells were isolated from preantral follicles (Diethylstilbestrol-primed 21-day old rats; 1 mg/d, subcutaneous injection for 3 consecutive days) and antral follicles (Equine chorionic gonadotropin –injected 22-day old rats; 10 IU intraperitoneal injection; animal sacrificed 48 h post-treatment). Granulosa cells were cultured with DHT (0 and 1 µM) for 24 h and 36 h.

## Slide 5
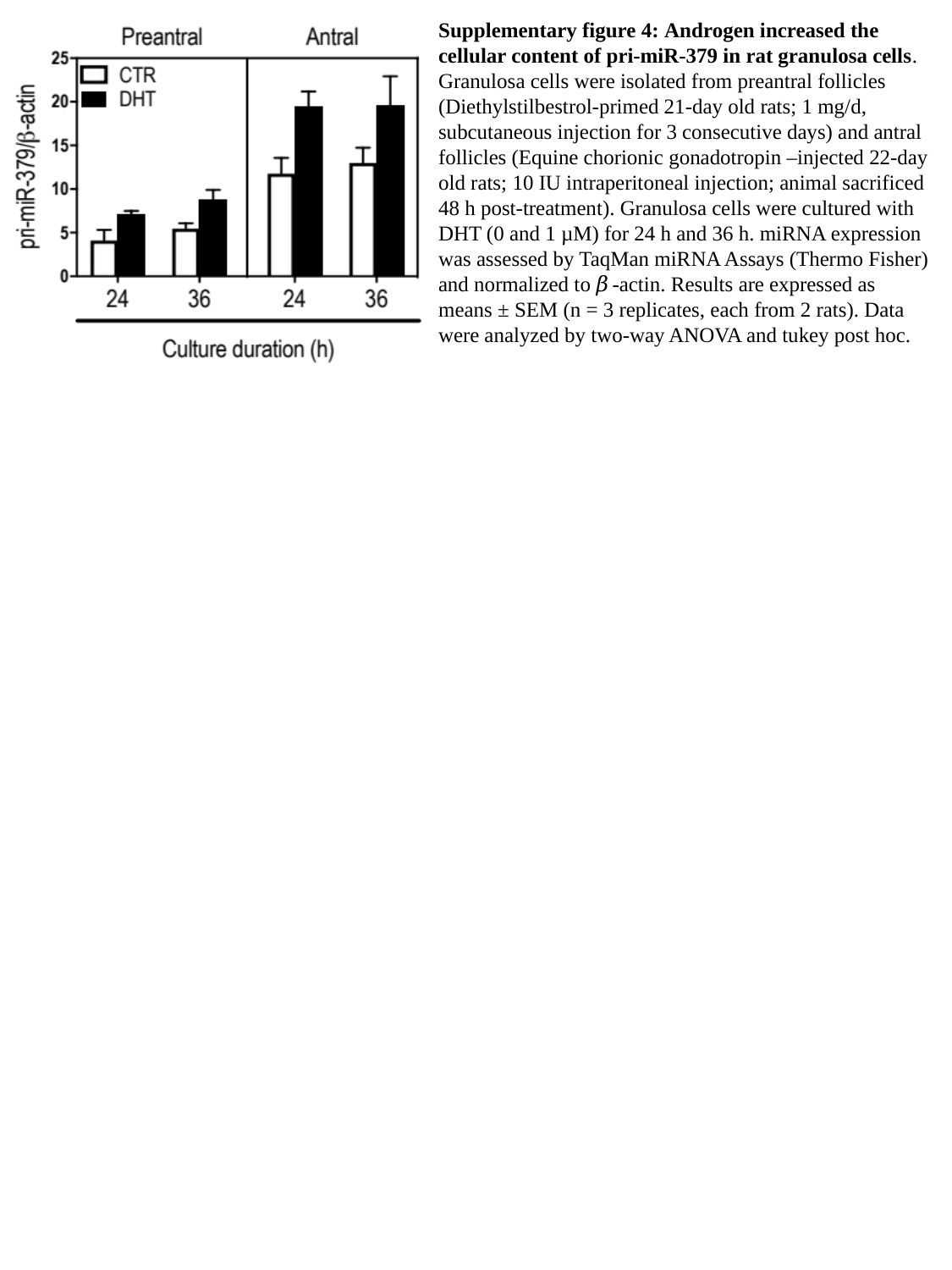

Supplementary figure 4: Androgen increased the cellular content of pri-miR-379 in rat granulosa cells. Granulosa cells were isolated from preantral follicles (Diethylstilbestrol-primed 21-day old rats; 1 mg/d, subcutaneous injection for 3 consecutive days) and antral follicles (Equine chorionic gonadotropin –injected 22-day old rats; 10 IU intraperitoneal injection; animal sacrificed 48 h post-treatment). Granulosa cells were cultured with DHT (0 and 1 µM) for 24 h and 36 h. miRNA expression was assessed by TaqMan miRNA Assays (Thermo Fisher) and normalized to 𝛽-actin. Results are expressed as means ± SEM (n = 3 replicates, each from 2 rats). Data were analyzed by two-way ANOVA and tukey post hoc.

## Slide 6
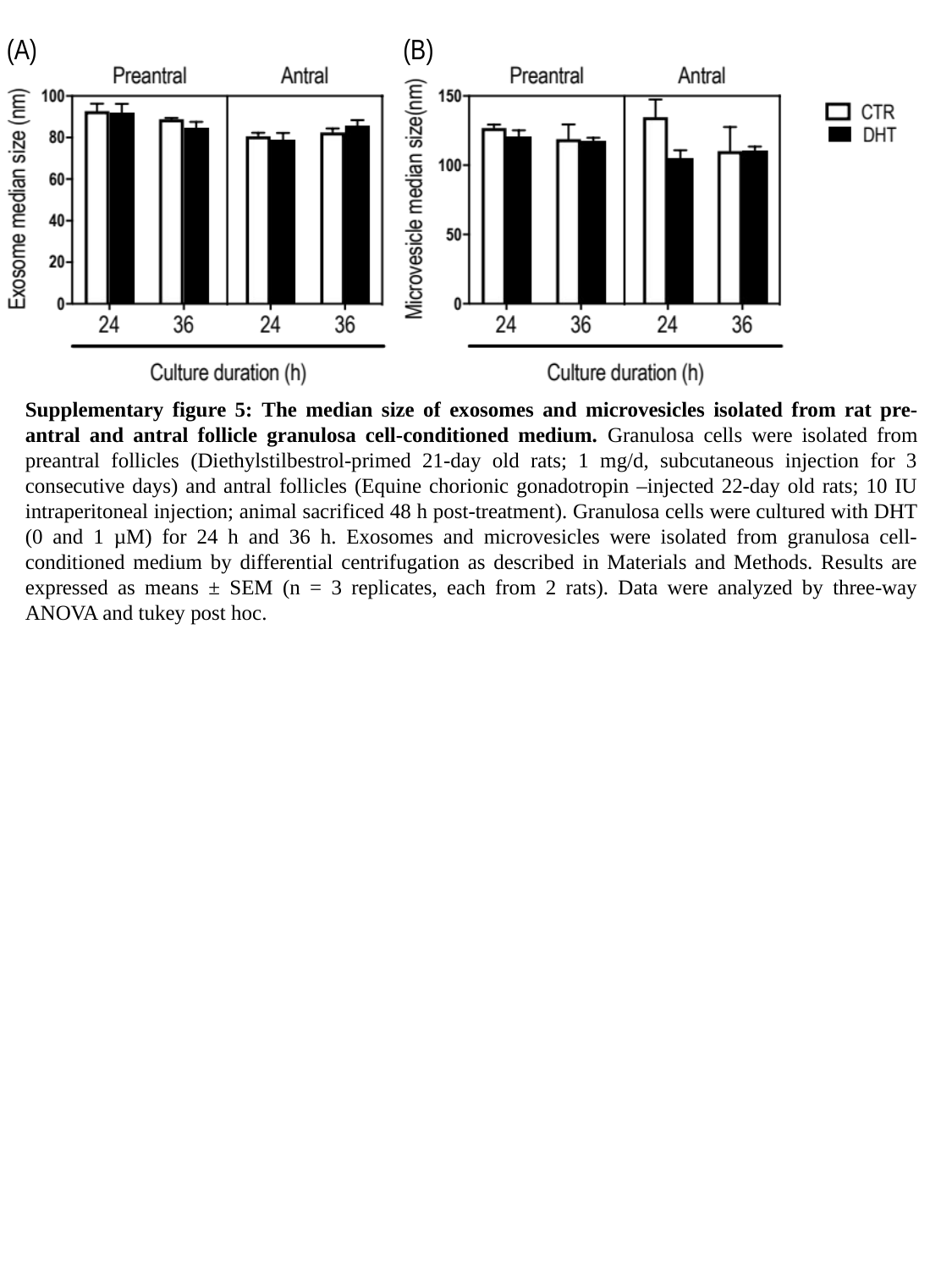

(A)
(B)
Supplementary figure 5: The median size of exosomes and microvesicles isolated from rat pre-antral and antral follicle granulosa cell-conditioned medium. Granulosa cells were isolated from preantral follicles (Diethylstilbestrol-primed 21-day old rats; 1 mg/d, subcutaneous injection for 3 consecutive days) and antral follicles (Equine chorionic gonadotropin –injected 22-day old rats; 10 IU intraperitoneal injection; animal sacrificed 48 h post-treatment). Granulosa cells were cultured with DHT (0 and 1 µM) for 24 h and 36 h. Exosomes and microvesicles were isolated from granulosa cell-conditioned medium by differential centrifugation as described in Materials and Methods. Results are expressed as means ± SEM (n = 3 replicates, each from 2 rats). Data were analyzed by three-way ANOVA and tukey post hoc.

## Slide 7
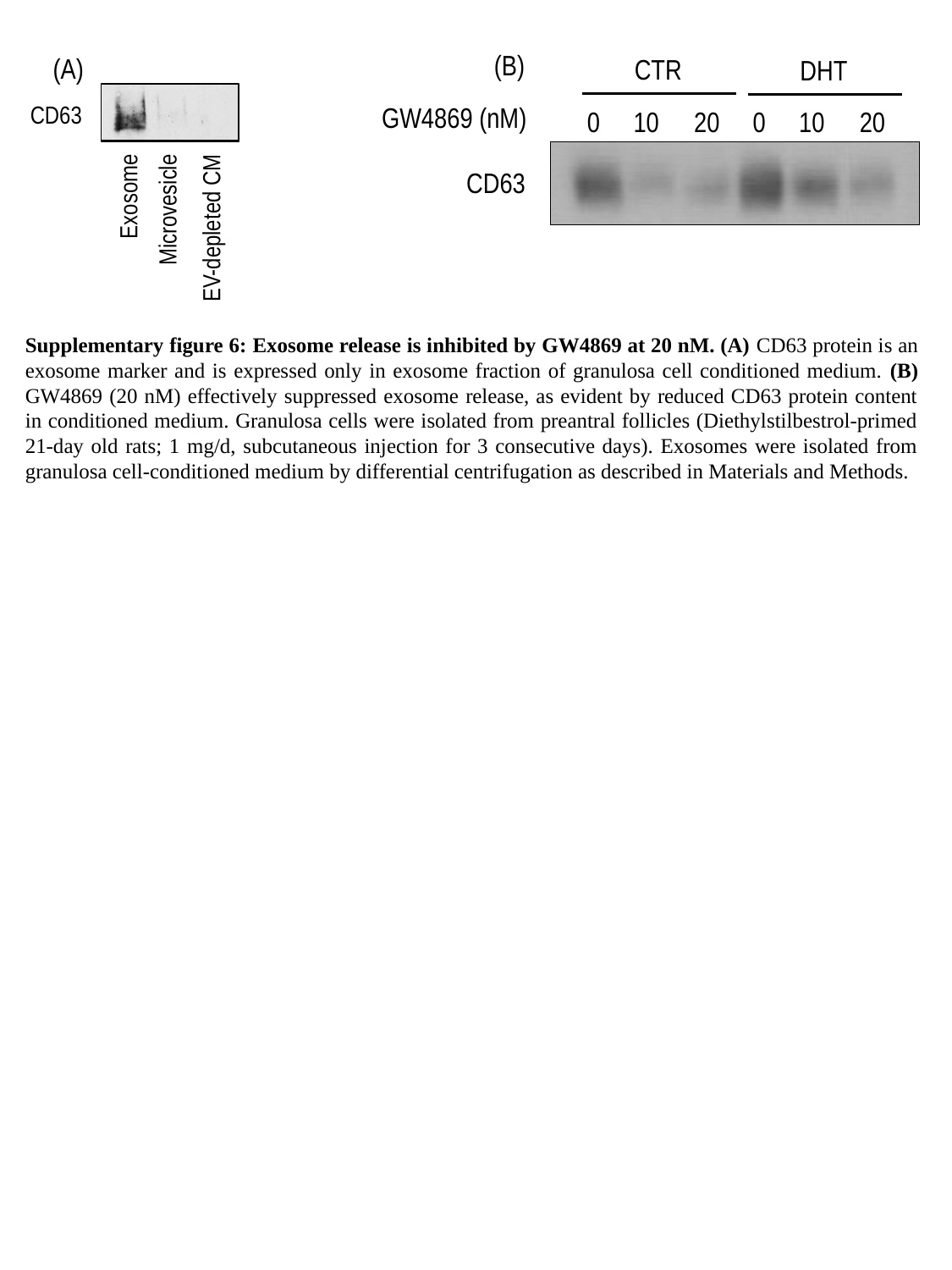

(B)
(A)
CTR
DHT
CD63
GW4869 (nM)
0
10
20
0
10
20
CD63
Exosome
Microvesicle
EV-depleted CM
Supplementary figure 6: Exosome release is inhibited by GW4869 at 20 nM. (A) CD63 protein is an exosome marker and is expressed only in exosome fraction of granulosa cell conditioned medium. (B) GW4869 (20 nM) effectively suppressed exosome release, as evident by reduced CD63 protein content in conditioned medium. Granulosa cells were isolated from preantral follicles (Diethylstilbestrol-primed 21-day old rats; 1 mg/d, subcutaneous injection for 3 consecutive days). Exosomes were isolated from granulosa cell-conditioned medium by differential centrifugation as described in Materials and Methods.

## Slide 8
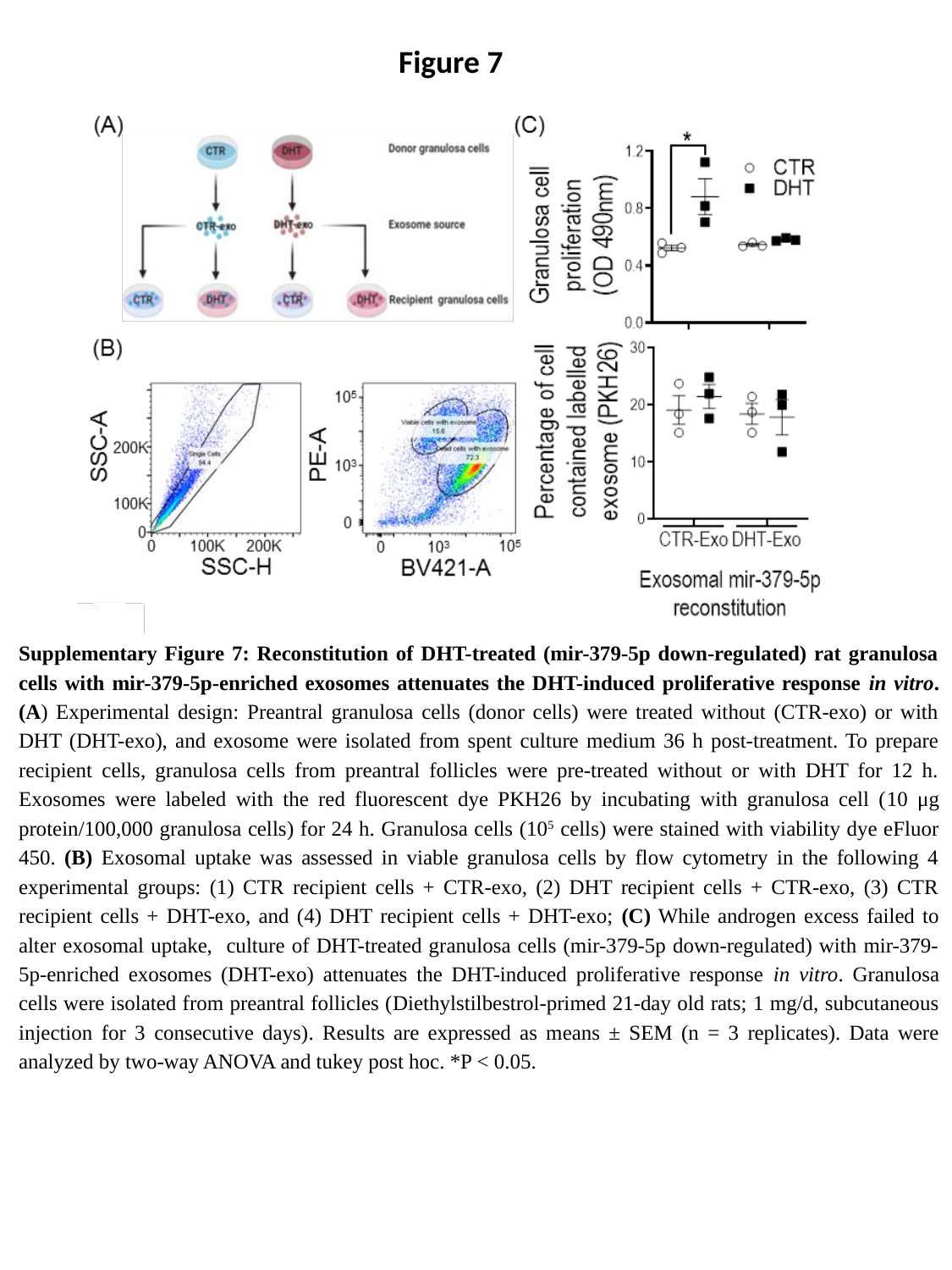

Figure 7
Supplementary Figure 7: Reconstitution of DHT-treated (mir-379-5p down-regulated) rat granulosa cells with mir-379-5p-enriched exosomes attenuates the DHT-induced proliferative response in vitro. (A) Experimental design: Preantral granulosa cells (donor cells) were treated without (CTR-exo) or with DHT (DHT-exo), and exosome were isolated from spent culture medium 36 h post-treatment. To prepare recipient cells, granulosa cells from preantral follicles were pre-treated without or with DHT for 12 h. Exosomes were labeled with the red fluorescent dye PKH26 by incubating with granulosa cell (10 μg protein/100,000 granulosa cells) for 24 h. Granulosa cells (105 cells) were stained with viability dye eFluor 450. (B) Exosomal uptake was assessed in viable granulosa cells by flow cytometry in the following 4 experimental groups: (1) CTR recipient cells + CTR-exo, (2) DHT recipient cells + CTR-exo, (3) CTR recipient cells + DHT-exo, and (4) DHT recipient cells + DHT-exo; (C) While androgen excess failed to alter exosomal uptake, culture of DHT-treated granulosa cells (mir-379-5p down-regulated) with mir-379-5p-enriched exosomes (DHT-exo) attenuates the DHT-induced proliferative response in vitro. Granulosa cells were isolated from preantral follicles (Diethylstilbestrol-primed 21-day old rats; 1 mg/d, subcutaneous injection for 3 consecutive days). Results are expressed as means ± SEM (n = 3 replicates). Data were analyzed by two-way ANOVA and tukey post hoc. *P < 0.05.

## Slide 9
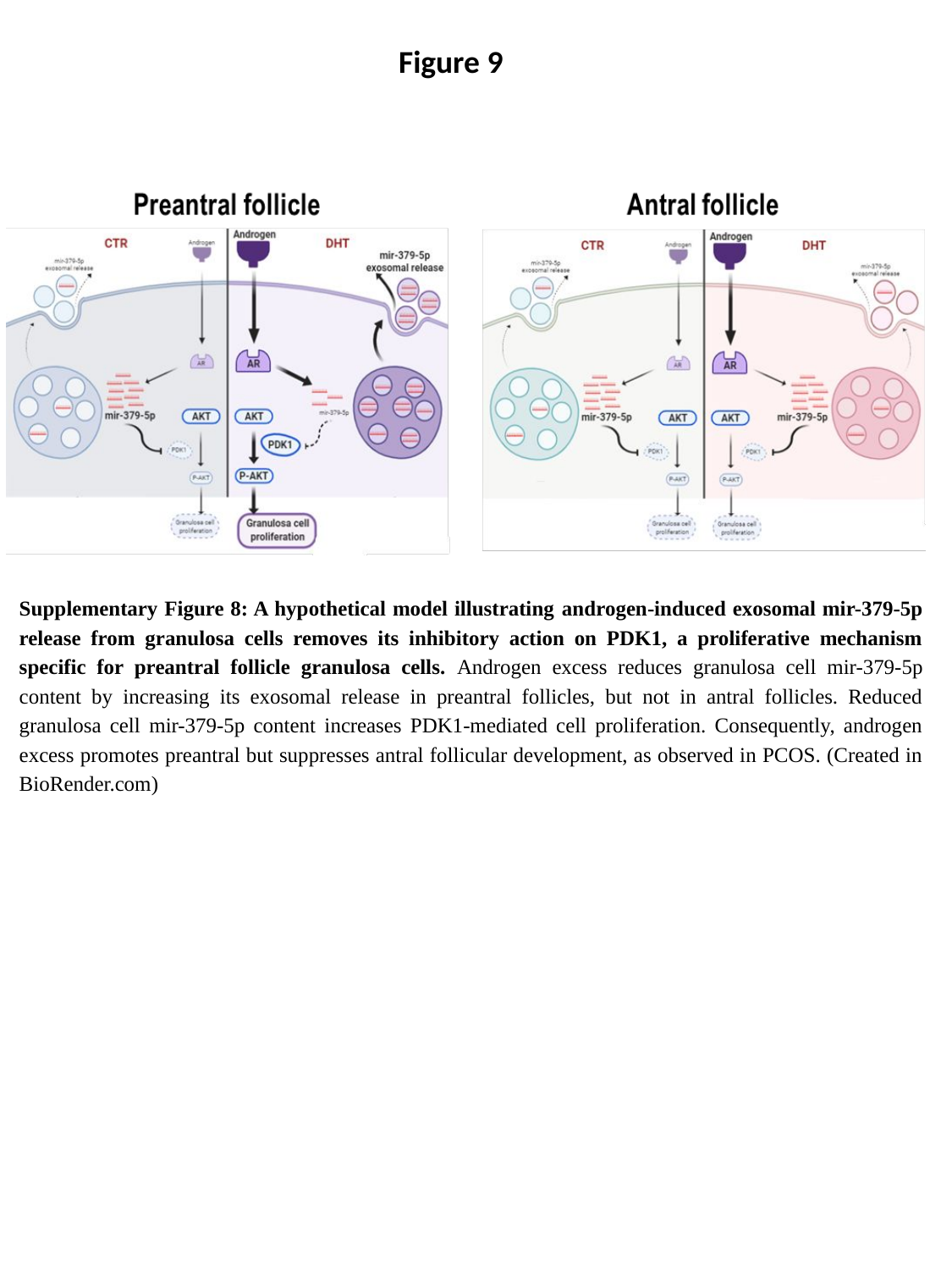

Figure 9
Supplementary Figure 8: A hypothetical model illustrating androgen-induced exosomal mir-379-5p release from granulosa cells removes its inhibitory action on PDK1, a proliferative mechanism specific for preantral follicle granulosa cells. Androgen excess reduces granulosa cell mir-379-5p content by increasing its exosomal release in preantral follicles, but not in antral follicles. Reduced granulosa cell mir-379-5p content increases PDK1-mediated cell proliferation. Consequently, androgen excess promotes preantral but suppresses antral follicular development, as observed in PCOS. (Created in BioRender.com)

## Slide 10
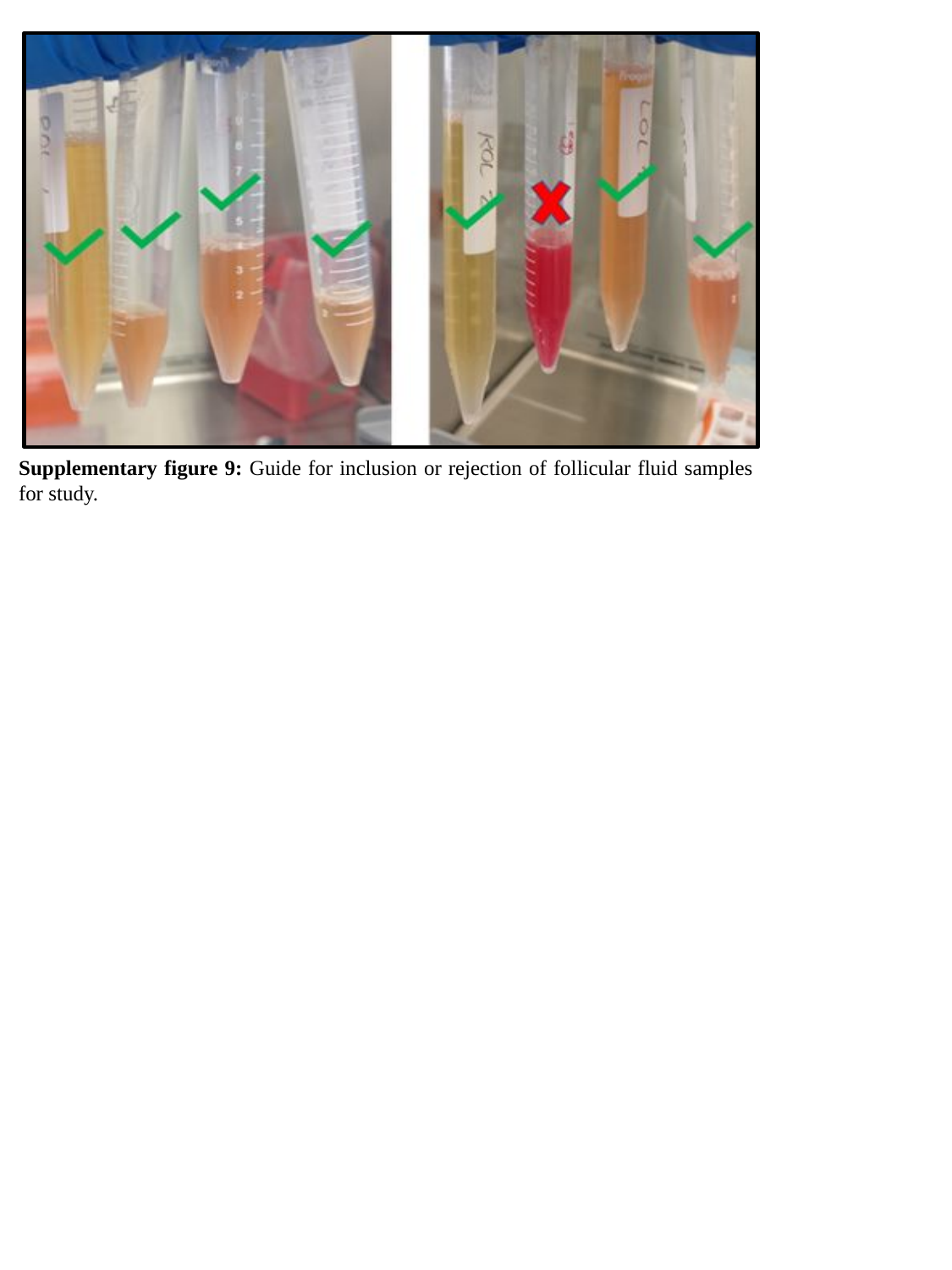

Supplementary figure 9: Guide for inclusion or rejection of follicular fluid samples for study.

## Slide 11
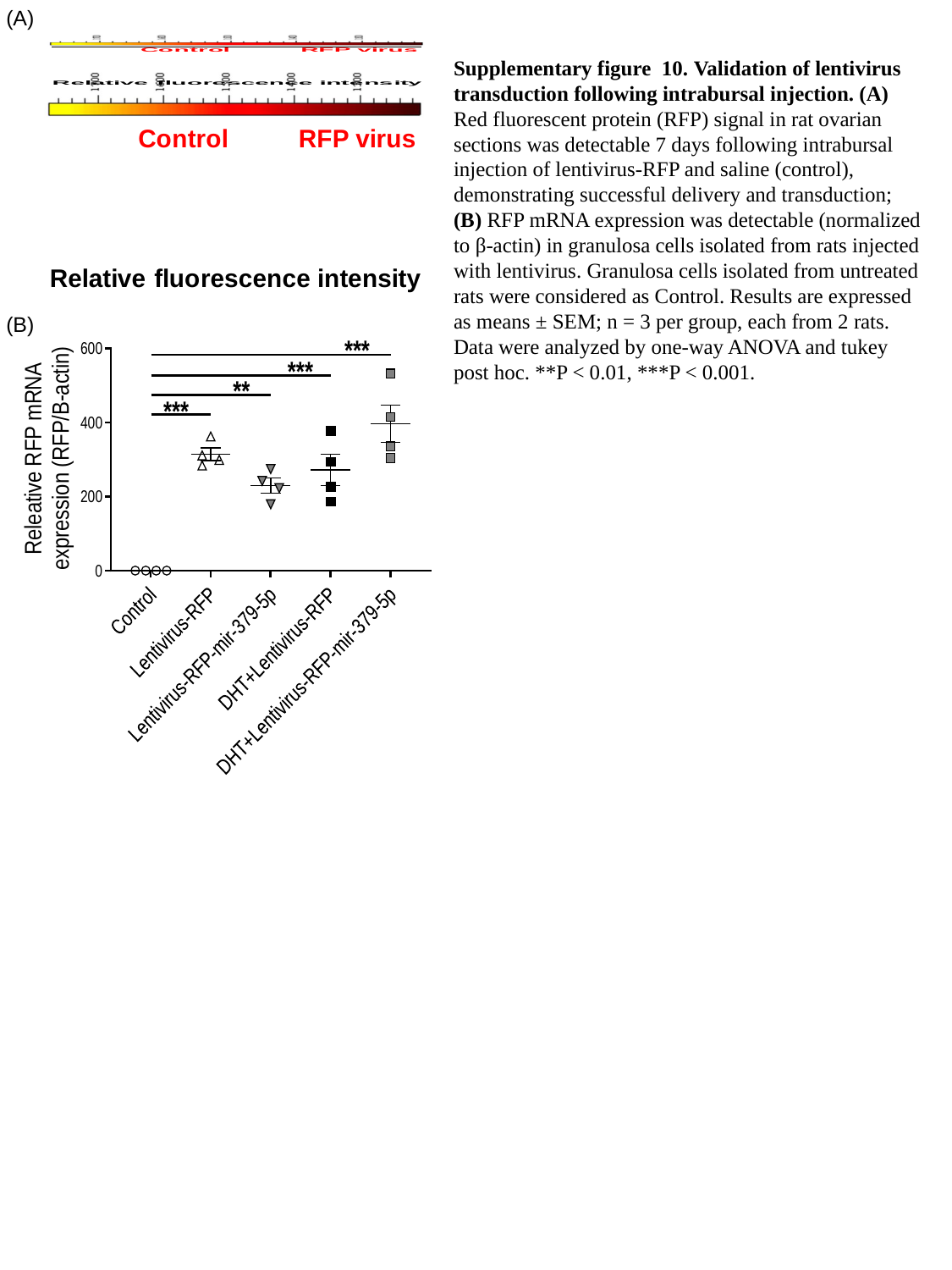

(A)
Supplementary figure 10. Validation of lentivirus transduction following intrabursal injection. (A) Red fluorescent protein (RFP) signal in rat ovarian sections was detectable 7 days following intrabursal injection of lentivirus-RFP and saline (control), demonstrating successful delivery and transduction; (B) RFP mRNA expression was detectable (normalized to β-actin) in granulosa cells isolated from rats injected with lentivirus. Granulosa cells isolated from untreated rats were considered as Control. Results are expressed as means ± SEM; n = 3 per group, each from 2 rats. Data were analyzed by one-way ANOVA and tukey post hoc. **P < 0.01, ***P < 0.001.
(B)

## Slide 12
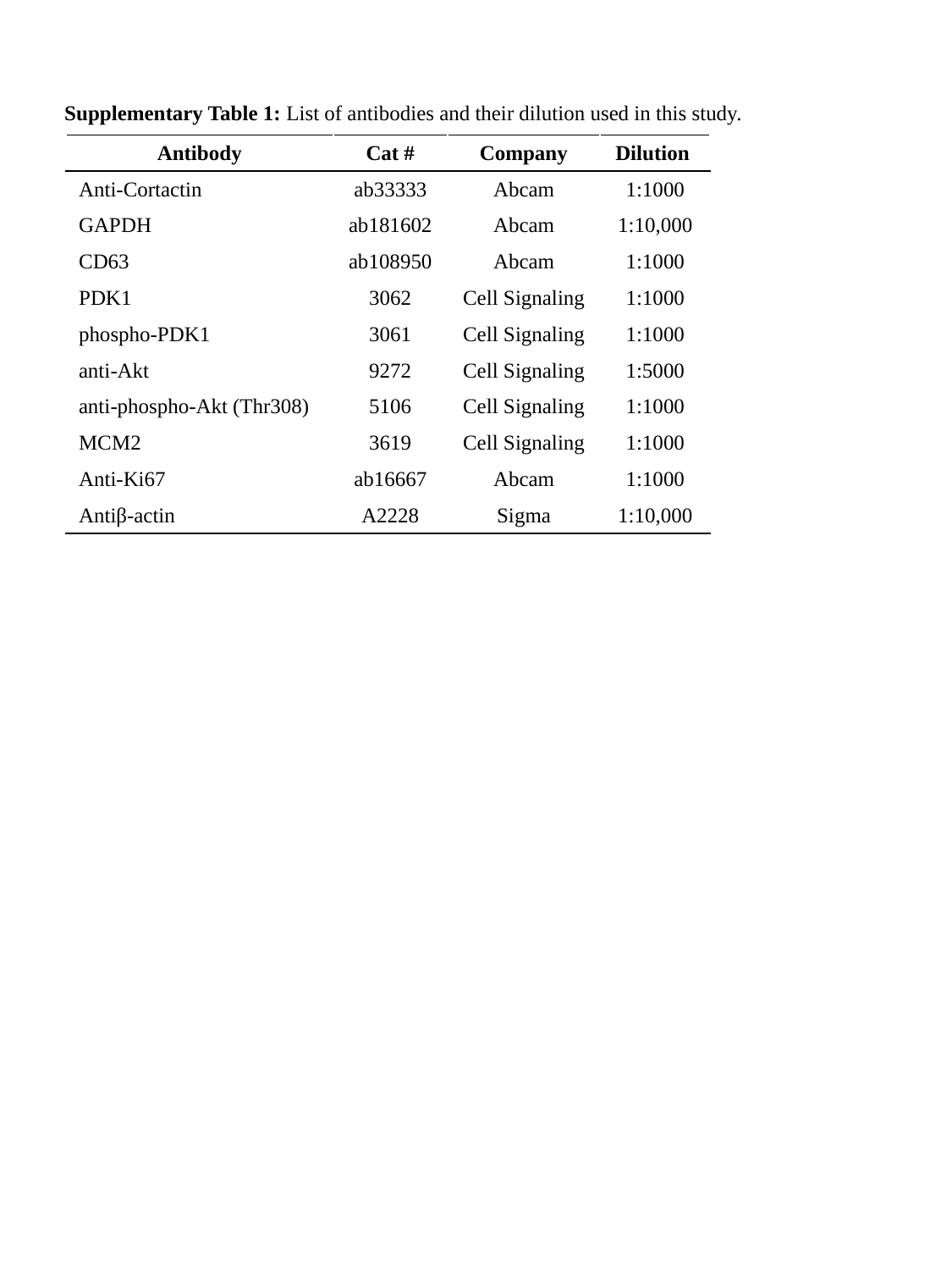

Supplementary Table 1: List of antibodies and their dilution used in this study.
| Antibody | Cat # | Company | Dilution |
| --- | --- | --- | --- |
| Anti-Cortactin | ab33333 | Abcam | 1:1000 |
| GAPDH | ab181602 | Abcam | 1:10,000 |
| CD63 | ab108950 | Abcam | 1:1000 |
| PDK1 | 3062 | Cell Signaling | 1:1000 |
| phospho-PDK1 | 3061 | Cell Signaling | 1:1000 |
| anti-Akt | 9272 | Cell Signaling | 1:5000 |
| anti-phospho-Akt (Thr308) | 5106 | Cell Signaling | 1:1000 |
| MCM2 | 3619 | Cell Signaling | 1:1000 |
| Anti-Ki67 | ab16667 | Abcam | 1:1000 |
| Antiβ-actin | A2228 | Sigma | 1:10,000 |

## Slide 13
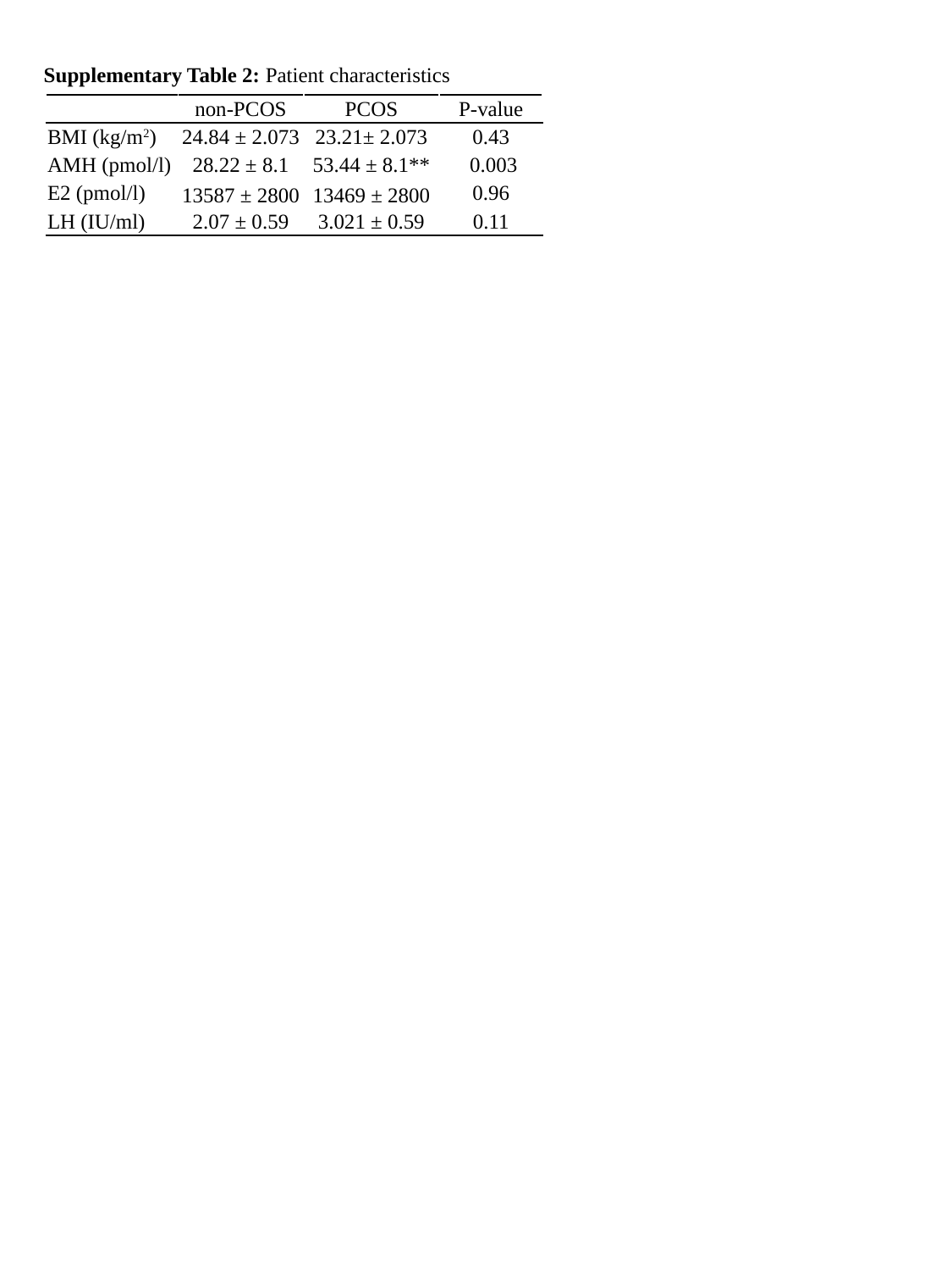

Supplementary Table 2: Patient characteristics
| | non-PCOS | PCOS | P-value |
| --- | --- | --- | --- |
| BMI (kg/m2) | 24.84 ± 2.073 | 23.21± 2.073 | 0.43 |
| AMH (pmol/l) | 28.22 ± 8.1 | 53.44 ± 8.1\*\* | 0.003 |
| E2 (pmol/l) | 13587 ± 2800 | 13469 ± 2800 | 0.96 |
| LH (IU/ml) | 2.07 ± 0.59 | 3.021 ± 0.59 | 0.11 |

## Slide 14
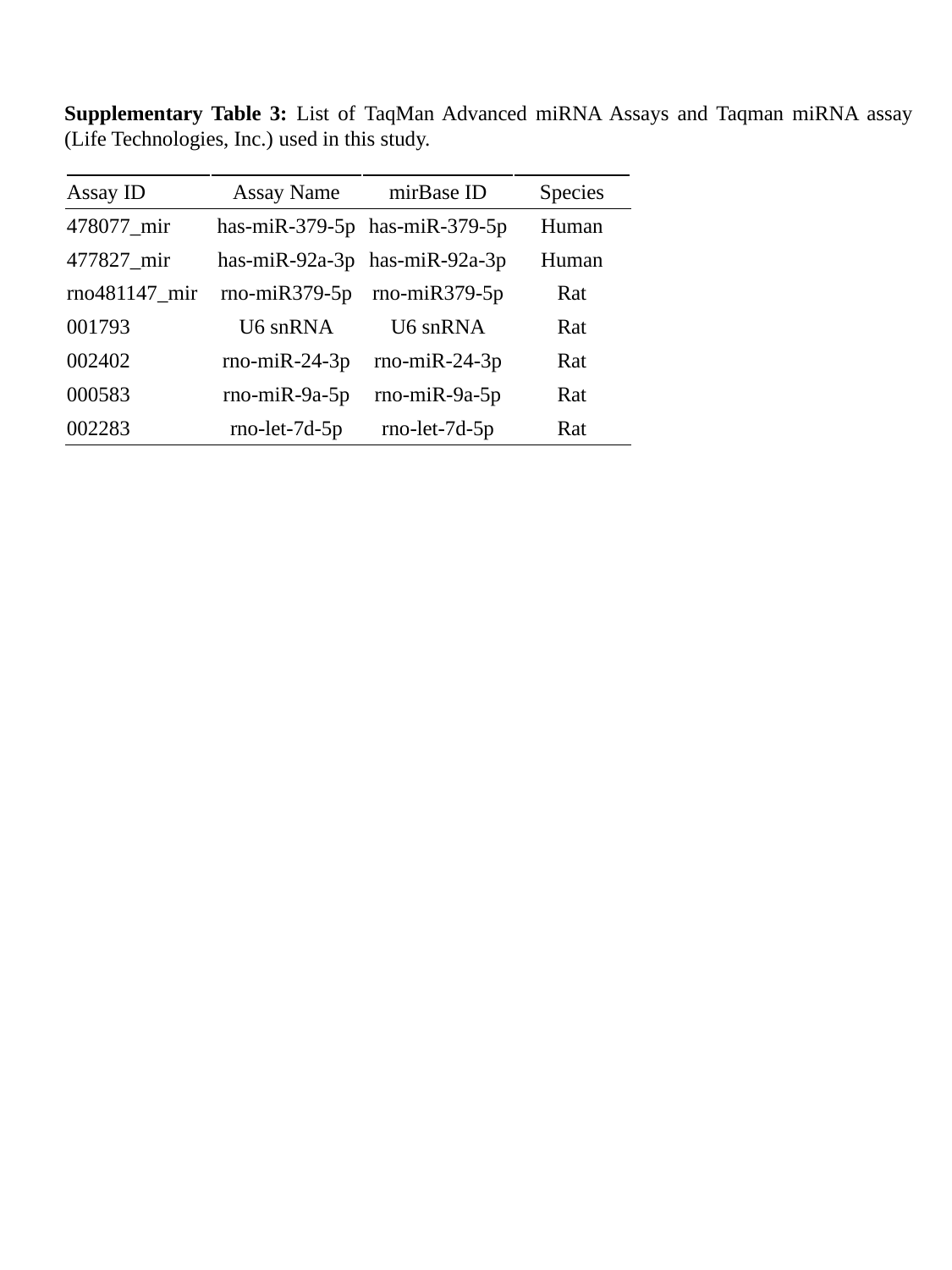

Supplementary Table 3: List of TaqMan Advanced miRNA Assays and Taqman miRNA assay (Life Technologies, Inc.) used in this study.
| Assay ID | Assay Name | mirBase ID | Species |
| --- | --- | --- | --- |
| 478077\_mir | has-miR-379-5p | has-miR-379-5p | Human |
| 477827\_mir | has-miR-92a-3p | has-miR-92a-3p | Human |
| rno481147\_mir | rno-miR379-5p | rno-miR379-5p | Rat |
| 001793 | U6 snRNA | U6 snRNA | Rat |
| 002402 | rno-miR-24-3p | rno-miR-24-3p | Rat |
| 000583 | rno-miR-9a-5p | rno-miR-9a-5p | Rat |
| 002283 | rno-let-7d-5p | rno-let-7d-5p | Rat |
